# A Diagnosis-Based Siamese Network for Fault Detection Through Transfer Learning

**DOI:** 10.1021/acs.jcim.5c00809

**Published:** 2025-06-30

**Authors:** João G. Neto, Karla Figueiredo, João B. P. Soares, Amanda L. T. Brandão

**Affiliations:** † Department of Chemical and Materials Engineering, Pontifical Catholic University of Rio de Janeiro, 225, Marquês de São Vicente Street, Gávea, Rio de Janeiro, RJ 22451-900, Brazil; ‡ Department of Computer Science, Rio de Janeiro State University, 524, Rector João Lyra Filho Pavilion, sixth floor, Maracanã, Rio de Janeiro, RJ 20550-013, Brazil; § Department of Chemical Engineering, University of Alberta, 9211,116 Street, Edmonton, Alberta T6G 1H9, Canada

## Abstract

Traditional deep-learning-based approaches often struggle with data imbalance and variability across fault conditions and normal scenarios, especially in industrial processes. Besides, inconsistent feature distributions from combining different fault conditions into the same category are a limitation for many data-driven algorithms. This study proposes a fault detection framework that combines Siamese neural networks with transfer learning, using a pretrained fault diagnosis model as its backbone, taking advantage of knowledge related to the attribute space that characterizes individual fault patterns. Our method transforms the detection classification problem into an embedding similarity task, allowing for improved differentiation between normal and faulty operations. This approach poses an alternative for data imbalance and a lack of labeled anomaly data, as it is based on the combination of normal and faulty time series. Our best model achieved an F1-score of 91.41% on the test set, and the t-distributed stochastic neighbor embedding indicates that the knowledge transferred from diagnosis allowed the detection model to generate embeddings that discriminate between most faulty conditions. When analyzing individual fault detection rates, we observed that our model demonstrated superior performance compared with recent literature for most fault cases.

## Graphical abstract

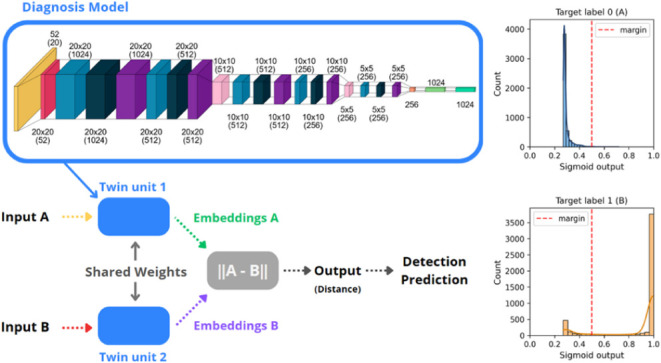

## Introduction

Fault Detection and Diagnosis (FDD) is important in maintaining efficient productivity and safety of industrial processes. Due to their complexity, such systems are prone to anomalies and faults, meaning that detection is essential for mitigating risks and maintaining process reliability.^1–5^ Traditional methods for FDD often rely on statistical techniques or analytical modeling, which may struggle with nonlinearities and high-dimensional data.^6^

Recent advancements in Deep Learning (DL) have provided promising alternatives to these challenges.^7–9^ However, these approaches heavily depend on abundant and high-quality data, which include a balanced distribution of fault samples.^10^ Furthermore, in industrial settings, systems typically operate under normal conditions, thereby reducing the likelihood of records of faulty behavior. This scarcity can limit the ability of data-driven FDD techniques to capture critical fault characteristics. Consequently, implementing these methods can be unviable if this problem is not addressed first.^11,12^

Imbalanced data sets are a common scenario in multiclass classification problems. In fact, there are well-established oversampling and undersampling techniques for data balancing. Nevertheless, these methods can introduce noise into the data set, potentially leading to overfitting in the case of oversampling or loss of valuable information when undersampling is applied. As a result, the model’s generalization performance may be compromised, affecting its ability to make accurate predictions on unseen data. Besides, time-series problems require more advanced applications regarding balancing techniques.^12–14^

Another alternative is the use of classical modeling techniques to generate synthetic data. Simulation-based approaches create new data points that adhere to the statistical properties of the original data once the model is validated. For instance, the Monte Carlo method and domain-specific mathematical systems of equations can represent chemical processes realistically.^15–21^ In time-series problems, these techniques help maintain temporal dependencies and structural patterns. A notable example is the use of numerical simulation-based models for fault diagnosis, as introduced by Xiang and Zhong (2016).^22^ Their work focused on diagnosing faults in rotating machinery by using FEM-based vibration signal simulation and classification. Consequently, several FDD benchmark data sets were partially or fully developed using simulations, providing standardized testing grounds for evaluating classification models in imbalanced scenarios.^23^

The Tennessee Eastman Process (TEP) is a widely used benchmark that allows researchers to compare different methodologies under controlled conditions. This data set provides a realistic simulation of complex chemical processes, enabling the systematic introduction and analysis of various fault scenarios. By offering a risk-free environment that mimics real-world challenges, TEP supports the development and evaluation of advanced FDD algorithms. Its diverse fault types, including operational disturbances and sensor failures, make it a valuable reference for assessing diagnostic approaches.^24–28^ These qualities make this data set suitable to use as our study case.

FDD models are commonly developed by attempting simultaneous detection and diagnosis or treating these aspects sequentially. In the first option, normal behavior is usually a class among different faults, while in the second, detection and diagnosis are studied independently, with the possibility of later aggregation. In either case, several approaches are possible, such as an ensemble of specialized models, hierarchized models, or a generalistic multiclass estimator.^8,9,29–31^

Specifically in detection models, grouping faulty operations into a single class is standard practice, simplifying the system into a binary classification problem. However, combining multiple infrequent classes into a single category can lead to poor classification performance due to inconsistent feature distributions.^32^ This study explored this issue by combining two state-of-the-art techniques: Siamese neural network (SNN) architecture and transfer learning.

SNNs are particularly well-suited for tasks that require similarity assessments,^33,34^ making them a natural choice for detecting deviations from normal operating states. Additionally, the contrastive loss penalizes mistakes based on the distance between the embedding representations of inputs.^35^ Consequently, the model has the flexibility to learn different feature distributions of the detection task independently from the differences between the varied faulty scenarios, as long as they are distinguishable from normal behavior.

While SNNs have been explored in some industrial contexts, their application to chemical process monitoring remains limited. For example, Takimoto et al. (2022)^36^ proposed a SNN-based anomaly detection method enhanced with an attention mechanism for visual inspection tasks in manufacturing. Their approach effectively handled few-shot scenarios, demonstrating that SNNs can be used in industrial settings, where abnormal data are scarce. However, to the best of our knowledge, such techniques have not yet been extended to chemical plant environments that involve different types of data and fault characteristics. This gap highlights the novelty of our work in applying SNN-based models to fault detection in chemical processes.

Since SNNs comprise a macroarchitecture that supports a substructure, we used transfer learning in our investigation. In other words, we explored models that start with knowledge about embeddings from each fault by using the best-performing CNN model from our previous TEP fault diagnosis study^37^ as their backbone. Our approach reverses the conventional order of detection preceding diagnosis, providing a new perspective for developing FDD solutions.

The remainder of this article is organized as follows: the section Theorectical Framework provides the theoretical framework necessary to understand the techniques adopted in our study; the section Methodology describes the steps of the investigation, including data pretreatment, model architecture, fine-tuning, and evaluation methods; the section Results and Discussion critically assesses and compares our results with recent literature; and finally, the section Conclusions summarizes our findings and indicates possible directions for future studies.

## Theoretical Framework

This section provides an overview of Siamese neural networks, their fundamental architecture, and their advantages for fault detection tasks. Additionally, it briefly introduces the Tennessee Eastman Process and describes the data set version used in this study.

### Siamese Neural Networks

Siamese neural networks were first introduced by Bromley et al. in 1993 for text verification,^33^ which involved comparing two handwritten signatures to determine whether they belonged to the same individual or not. Since their inception, SNNs have gained widespread popularity due to their ability to evaluate the similarity between inputs. As a result, they have become a powerful tool for tasks ranging from identity verification to anomaly detection.^34,38,39^

SNNs possess several characteristics that make them particularly suited for fault detection tasks. Unlike traditional classification models, they can perform well with limited data, as they focus on learning relationships or similarities rather than explicit classes.^34,38^ Models based on this type of architecture tend to generalize well to unseen examples by learning a similarity function, making them versatile for new fault scenarios or operating conditions. Fault detection data sets often suffer from imbalanced data, in which normal behavior samples usually outnumber faulty ones. SNNs mitigate this challenge by comparing pairs of samples rather than relying on direct class labels, which increases the data set by performing different pair combinations.

Additionally, they can learn different feature distributions of subcases in the same class since the representation vector can point to different regions of the embedding space. In other words, these subcases can be placed at different locations while still resulting in a distance that correctly evaluates the similarity between the compared cases.

A typical SNN comprises two identical subnetworks called twin networks or twin units that share the same architecture and weights. Each subnetwork processes one of the two inputs in parallel and produces a feature representation for each. The model compares these embeddings by calculating a distance metric, such as Euclidean distance or cosine similarity, to determine the relationship between the inputs. During backward propagation, the algorithm uses the gradients from both inputs to update the shared weights.^33,34^ Figure 1 shows a graphic representation of a general SNN architecture.

**Figure 1:**
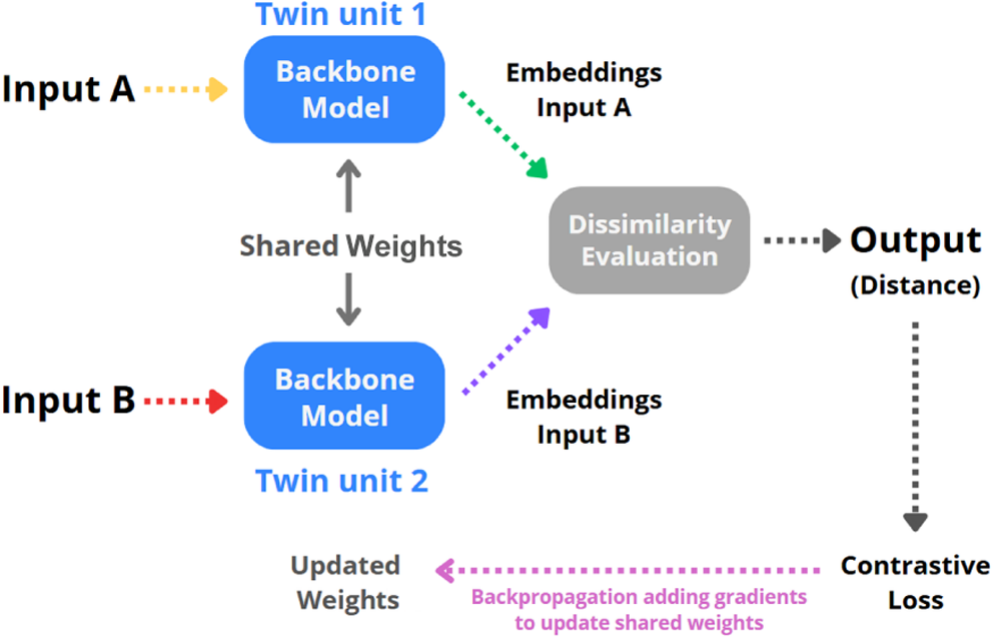
Diagram of an SNN framework.

The output of an SNN indicates a similarity measurement, which is not directly a classification. Thus, a loss function that connects such information to the correct label is required. This is the case of contrastive loss, as shown in eq 1.^35^

1 $$L=\frac{1}{N}\overset{N}{\underset{i=1}{\sum }}\left[\left(1-y_{i}\right)\times D^{2}+y_{i}\times \max{\left(0,m-D\right)}^{2}\right]$$

where *y* is the label for the pair, with zero for similar cases and one for dissimilar cases, *D* is the distance between the embeddings, *m* is a margin that defines the minimum distance required for dissimilar pairs, and *N* is the total number of samples in the set.

Any distance increases this loss by a quadratic factor when the target label is zero. By contrast, dissimilar cases result in a penalty only when the similarity measurement is between zero and the margin. In other words, this loss function enables the network to learn embeddings that minimize the distance for similar pairs and maximize the output for dissimilar pairs, considering a threshold and the target label. Therefore, the resulting model’s distance output is directly associated with the classification task and is equivalent to a label prediction through comparison with the margin.

SNNs have been applied in various univariate detection scenarios. For instance, they have been used to monitor bridge vibration patterns indicative of failure.^40^ In power systems, SNNs have been used to identify anomalies in operating conditions by monitoring current waveforms.^41^

### Tennessee Eastman Process Overview

The Tennessee Eastman Process is a simulation of an industrial chemical process, providing comprehensive data for process control and fault detection studies. Introduced by Downs and Vogel in 1993,^42^ the TEP models a dynamic chemical plant where multiple reactions interact to produce two primary products, as depicted in Figure 2. This simulation encompasses diverse features, including reaction kinetics, energy exchanges, and system disturbances, which collectively mirror the operational complexity of real-world chemical processes. The data set includes various measured and manipulated variables, presenting challenges and opportunities for testing advanced fault detection methodologies.

**Figure 2:**
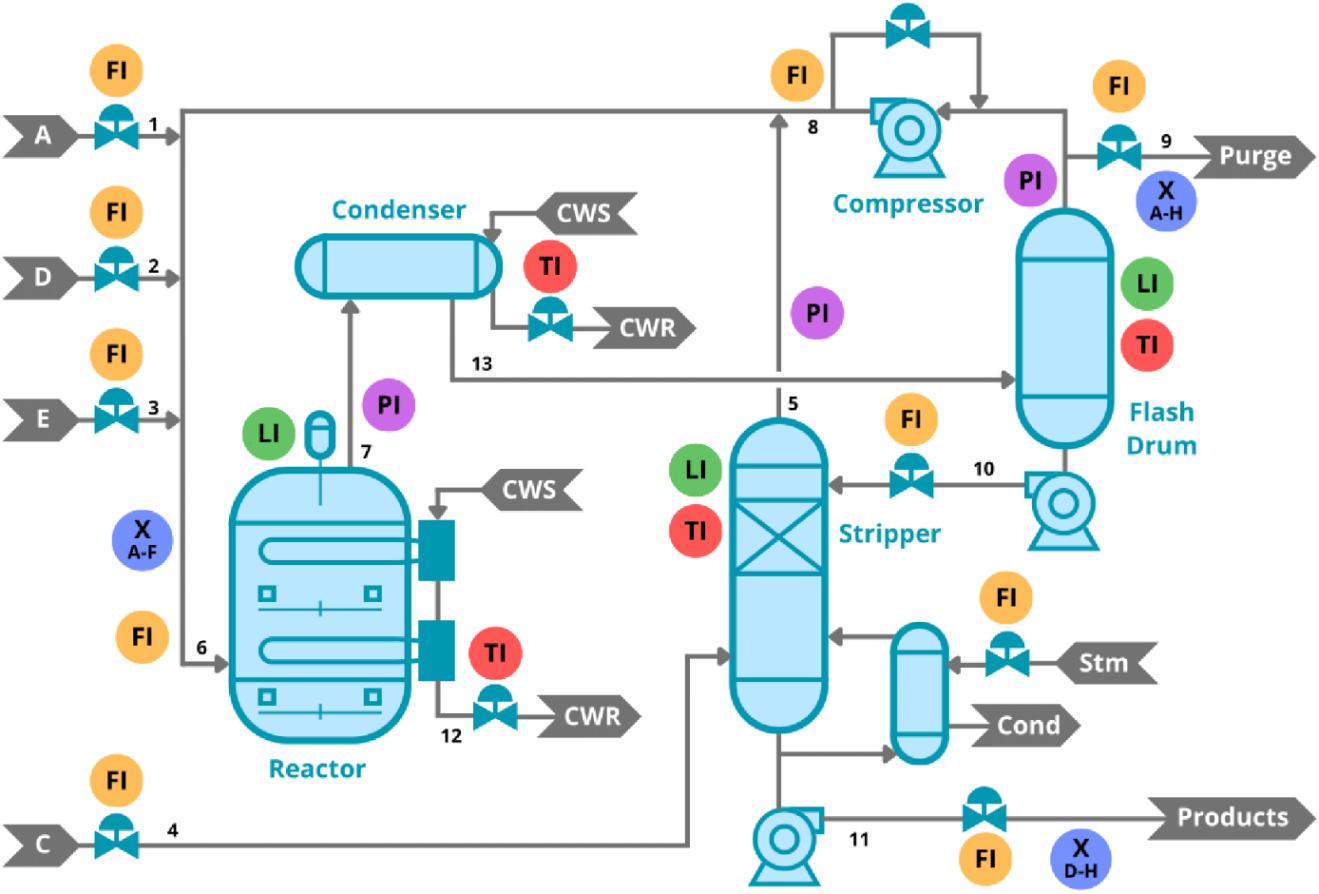
Diagram of the Tennessee Eastman Process. Reproduced from Neto, J. G.; Figueiredo, K.; Soares, J. B. P.; Brandão, A. L. T. Can Focusing on One Deep Learning Architecture Improve Fault Diagnosis Performance? *Journal of Chemical Information and Modeling* **2025**, 65, 1289–1304, DOI: 10.1021/acs.jcim.4c02060. Copyright 2025 American Chemical Society.

### Data and Software Availability

The data set utilized in this study is identical to that used in our previous work and is available on the Kaggle platform (https://www.kaggle.com/datasets/averkij/tennessee-eastman-process-simulation-dataset).^43^ However, the preprocessing procedure was modified to prevent data leakage and to modify the data format to make it compatible with the SNN input. This data set captures the behavior of a chemical system with four reactants (A, C, D, and E), an inert component (B), a byproduct (F), and two products (G and H). It consists of a total of 52 variables: 12 manipulated variables, 22 process measurements, and 18 component analyses. Fault scenarios include 20 distinct types, as summarized in Table 1. In our previous study, we identified faults 3, 9, 10, 15, and 16 as particularly difficult to diagnose compared to the others, which is in accordance with the literature.^37,44,45^ This data set does not include the 21st fault, which was introduced in newer versions of the data set.

**Table 1:** List and Description of Faults of the TEP

No.	Description	Type
1	A/C feed ratio, B composition constant (stream 4)	Step
2	B composition, A/C feed ratio constant (stream 4)	Step
3	D feed temperature (stream 2)	Step
4	Reactor cooling water inlet temperature	Step
5	Condenser cooling water inlet temperature	Step
6	A feed loss (stream 1)	Step
7	C header pressure loss-reduced availability (stream 4)	Step
8	A, B, and C feed composition (stream 4)	Random
9	D feed temperature (stream 2)	Random
10	C feed temperature (stream 4)	Random
11	Reactor cooling water inlet temperature	Random
12	Condenser cooling water inlet temperature	Random
13	Reaction kinetics	Slow drift
14	Reactor cooling water value	Sticking
15	Condenser cooling water value	Sticking
16	Unknown	Unknown
17	Unknown	Unknown
18	Unknown	Unknown
19	Unknown	Unknown
20	Unknown	Unknown

## Methodology

This section details the steps to preprocess data, investigate model architectures, train them under various configurations, and evaluate their performance. The study development was carried out in Python with the Pandas^46^ and Matplotlib^47^ libraries for data visualization. At the same time, TensorFlow,^48^ the Keras module of TensorFlow,^49^ and Scikit-learn^50^ were the tools used for data handling, model designing, training, and evaluation. Additionally, we executed all stages of the study on the same equipment with the following specifications: 32 GB RAM, i5-13600K CPU, and GeForce RTX 4070 Ti 12 GB.

### Data Pretreatment

The data for this investigation were a subsample of the same TEP data set we used in the fault diagnosis model.^37,43^ We exclusively used the data originally labeled training for both faulty and normal scenarios. Besides, we kept a 20-data-point observation window.

We structured the data in the expected format for a two-input SNN model. For better clarification, we refer to input one as the base time series and input two as the pair time series. Thus, a row of our data set contains one base time series and one pair time series grouped as inputs, followed by the target label.

In order to avoid data leakage, we removed the samples present in the training, validation, and testing sets of our previous diagnosis study before performing a new random subsampling from the TEP data set. All windows in the base time series correspond to normal behavior, ensuring that any detected fault originates from the pair time series. Additionally, we made sure to prevent the base time series from being compared to themselves in order to avoid trivial comparisons and ensure meaningful pairwise learning.

The primary goal of the fault evaluation system is to distinguish between normal and anomalous operations rather than to classify specific types of faults. Training on fault–fault pairs can lead to overfitting to intrafault similarities, which weakens the discriminative signal needed to effectively separate normal from abnormal behavior. In other words, fault–fault pairs induce the model to learn subtle variations within the same fault class rather than focusing on the critical differences between normal and anomalous behavior. Moreover, given the limited number of fault instances, the training strategy was focused on normal–normal and normal–fault pairs as these align more closely with the objectives of fault detection.

The selection of faulty time series was stratified to represent all types of faults in equal proportions. The target labels followed the pairing process depending on the resulting combination, with zero being similar and one being dissimilar. Additionally, we kept a balanced data set with 50% for each label.

Despite isolating time series between data sets, we performed a random selection with replacement within the same data set as long as the base-pair combination remained unique. Consequently, we created data sets consisting of 70,000 samples for training, 20,000 for validation, and 10,000 for testing. Figure 3 summarizes the data-structuring process.

**Figure 3:**
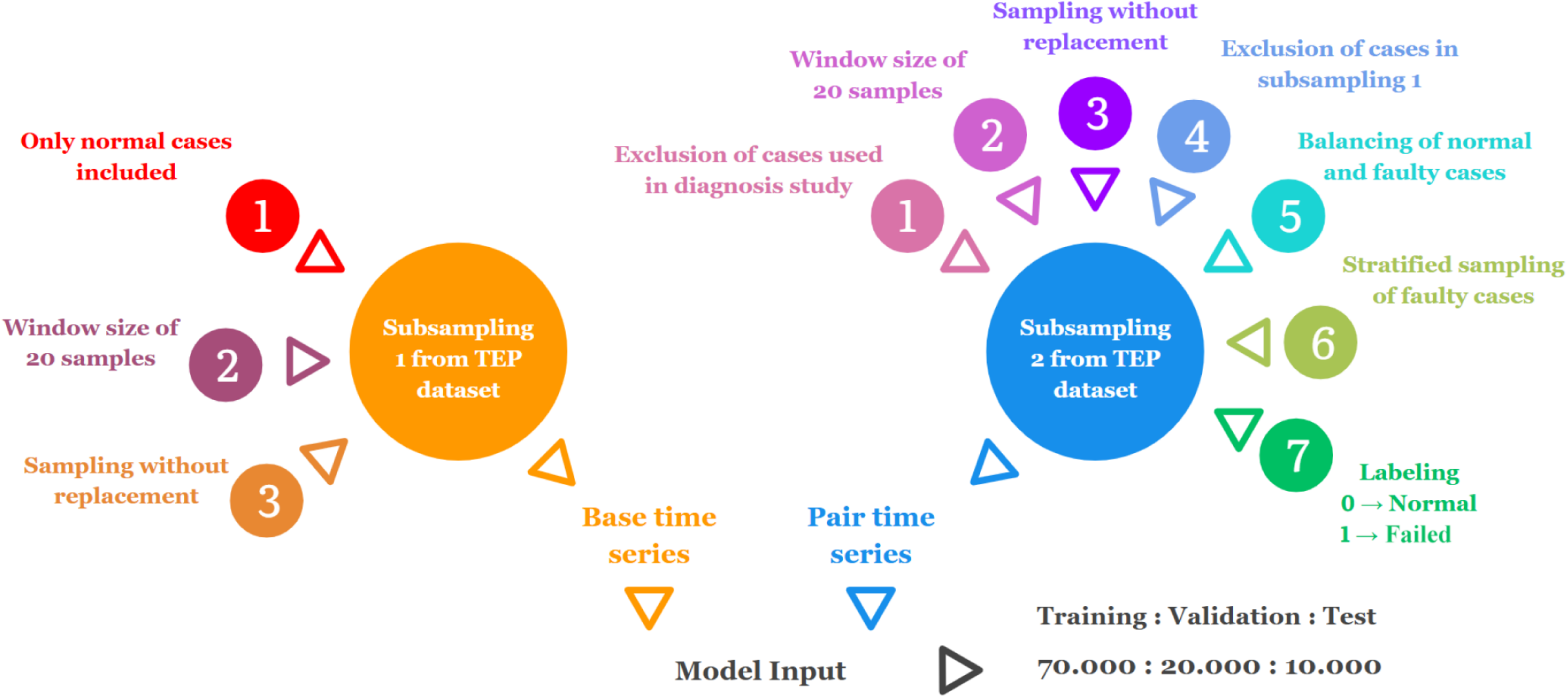
Summary of the data structuring process.

### SNN Architecture

The flexibility of the Siamese neural network framework allows suitable models to be used as its backbone, making it adaptable to different tasks. In this work, we applied transfer learning, a technique that uses a pretrained model as a starting point for a new but related task.^51^ By reusing and fine-tuning learned feature representations, transfer learning enables more efficient training, especially when labeled data are limited.

Transfer learning has been widely adopted in various machine learning domains due to its ability to mitigate data scarcity and distribution mismatches between training and target tasks.^51,52^ In this study, we explore the use of transfer learning not simply to reuse weights but to strategically inherit domain-specific representations from a pretrained fault diagnosis model. This approach is particularly relevant when the fault detection task shares underlying attribute space patterns with fault diagnosis. This enables better generalization and improved performance under fewer iterations, saving computational resources.

Diagnosis models are explicitly trained to differentiate among various fault types, making them more adept at capturing meaningful feature representations for each condition. These rich feature representations can be adapted through transfer learning for the detection task, improving the model’s ability to distinguish between normal and faulty states even for highly varied fault feature distributions. Additionally, since a pretrained diagnosis model already learned structured embeddings of fault conditions, the detection model benefits from a more informative input space, reducing the need for extensive labeled data and accelerating convergence during training. This approach effectively reframes detection as an embedding similarity problem rather than a direct classification task, aligning naturally with the Siamese neural network’s strengths. For these reasons, we selected a backbone model that had already demonstrated strong diagnostic performance in our previous study.^37^

Our previous model was derived from the model developed by Sun and Ren, 2021,^53^ which applies the Gramian Angular Summation Field (GASF) as part of the model architecture^54^ to the multivariate system of input time series, resulting in a collection of matrices, one for each variable. This transformation allowed us to apply feature learning through CNN processing. Figure 4 illustrates the base architecture that we used for the twin units.

**Figure 4:**
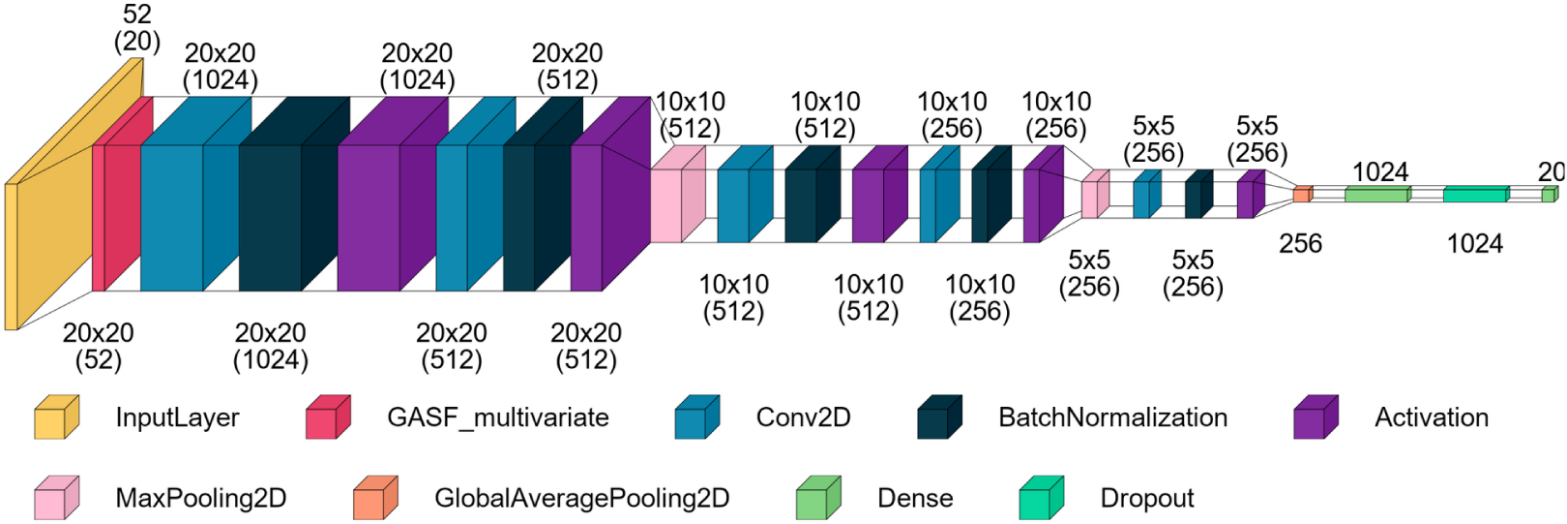
Best architecture from the diagnosis study. Reproduced from Neto, J. G.; Figueiredo, K.; Soares, J. B. P.; Brandão, A. L. T. Can Focusing on One Deep Learning Architecture Improve Fault Diagnosis Performance? *Journal of Chemical Information and Modeling* **2025**, 65, 1289–1304, DOI: 10.1021/acs.jcim.4c02060. Copyright 2025 American Chemical Society.

Since we wanted a rich representation of the system, we removed the Softmax layer, resulting in an embedding vector of 1024 elements to describe each input. Due to the high dimensionality and interpretability, we selected the Euclidean distance to compute the similarity between embeddings, which is directly compatible with the contrastive loss formulation applied in this study. While other metrics, such as cosine similarity, could also be considered, incorporating them would require reformulating the loss function, and this fell outside the scope of this investigation.

### Investigation Strategy

This section describes the investigation strategy used to refine the model. First, we focused on addressing the following question: ″How much data are enough?″ We evaluated the impact of the training data set size and explored key training parameters to establish a baseline. Then, we analyzed stability control: overfitting in the baseline, followed by underfitting in the stabilized model. Finally, we assessed the best-performing model through testing and comparison with the existing literature.

We trained all models in this study using the Adam optimizer with its default learning rate of 10^–3^ and contrastive loss with a margin of 1. Following our previous work, we conducted training by setting the batch size to 64 and a maximum of 500 epochs when applying early stopping. Additionally, we incorporated the Keras scheduler ReduceLROnPlateau with a reduction factor of 0.1 to dynamically adjust the learning rate.

In the first stage of our investigation, we examined the effect of training data set size on model performance using a holdout validation approach. Models were trained on progressively larger subsets, with 2.5% increments, until reaching the entire data set of 70,000 samples. Initially, we set the early stopping patience to 5 and the ReduceLROnPlateau patience to 4. However, evidence of premature stopping prompted a second phase of this stage, where models were trained with a fixed number of epochs to allow for better comparison. Since the most high-performing configurations completed training between 20 and 40 epochs for most cases, we selected the midpoint of this range (30 epochs) as a representative value for this phase to balance consistency and computational efficiency. This fixed epoch setup was used solely to support controlled comparisons across configurations and to inform adjustments to the early stopping and learning rate scheduler parameters in subsequent implementations. Based on the results from the second phase, we refined the patience values for early stopping and the learning rate scheduler to 15 and 10, respectively. We also included an analysis of the impact of the size of the training data set on the training duration to evaluate computational resources used. Finally, this stage was concluded with cross-validation using the selected configurations to establish a performance baseline for subsequent analyses. In all cross-validation cases in this study, we merged the training and validation sets and performed 5-fold cross-validation to assess model consistency.

In the second stage of our investigation, we aimed to address model stability and mitigate overfitting as the baseline model exhibited inconsistencies across cross-validation folds, an oscillatory behavior for the validation set, and a notable gap between training and validation performance. We froze all convolutional layers and conducted a new cross-validation under the same conditions because these behaviors could be related to the number of trainable parameters in the model. Since the baseline model already incorporated regularization strategies, we did not introduce additional regularization techniques at this stage.

Considering that freezing all convolutional layers solved the instability problem but also showed evidence of performance stagnation, the third stage focused on employing strategies against underfitting. We considered three parallel investigations to enhance the model performance, all using cross-validation to evaluate results. First, we reduced the dropout rate in the dense layer, evaluating models with a 30% dropout rate and no dropout, as the initial 50% dropout may have excessively regularized the model. Second, we introduced an additional dense layer, testing configurations with 1024 and 512 neurons, followed by a dropout layer with a 50% rate. All convolutional layers remained frozen during the dropout reduction and additional dense layer investigations. Third, in our transfer learning framework, certain groups of layers are frozen to preserve the learned feature representations from the source fault diagnosis model, while others remain trainable to allow adaptation to the target fault detection task. In order to investigate the balance between preserving diagnosis knowledge, model adaptability, and quantity of trainable parameters, we established four groups of convolutional layers to be set as frozen in independent models, as depicted in Figure 5. Each group always starts at the first convolutional layer of the structure and stops before the next convolutional or pooling layer after the last layer of the previous group. Consequently, we have a total of four models to analyze the effect of progressively freezing the transferred layers.

**Figure 5:**
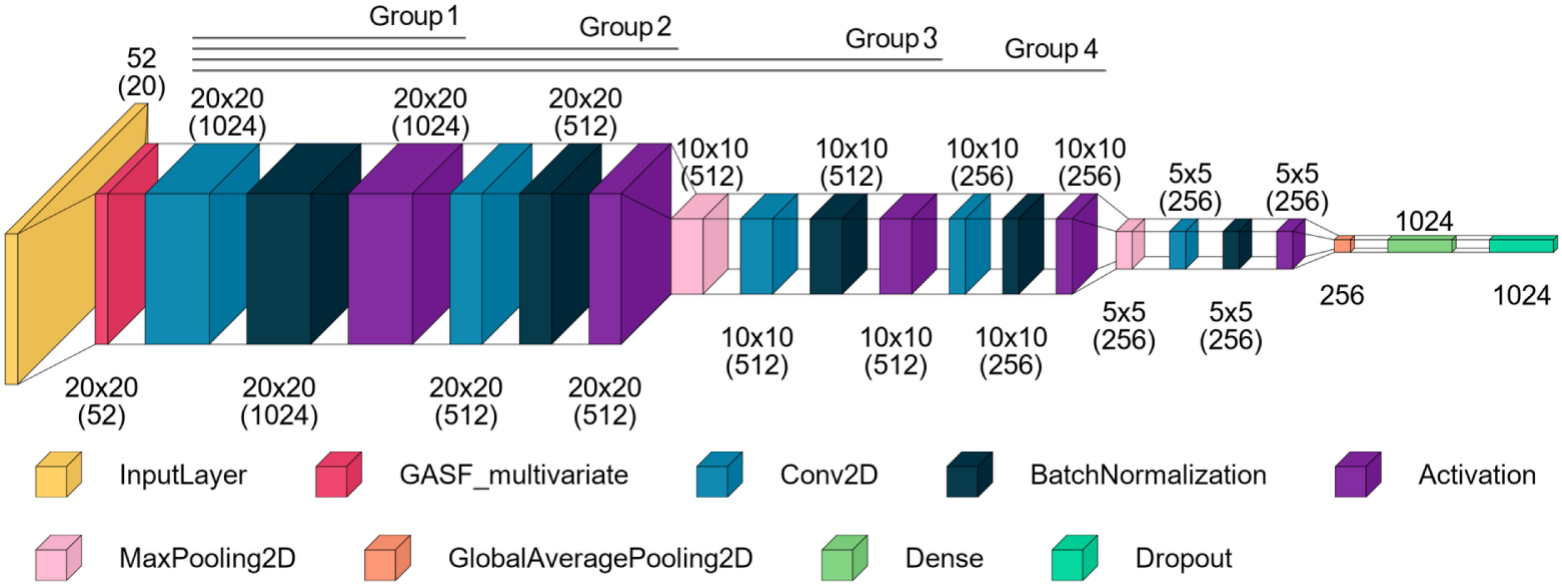
Groups of frozen convolutional layers in the twin unit. Reproduced from Neto, J. G.; Figueiredo, K.; Soares, J. B. P.; Brandão, A. L. T. Can Focusing on One Deep Learning Architecture Improve Fault Diagnosis Performance? *Journal of Chemical Information and Modeling* 2025, 65, 1289–1304, DOI: 10.1021/acs.jcim.4c02060. Copyright 2025 American Chemical Society.

In the final stage, we trained the best-performing configuration using the holdout method with the validation data set for early stopping and learning rate scheduling. We kept the last epoch state of trained models by setting the restore_best_weights parameter to false, as they consistently presented the best F1-score. The resulting model was then evaluated on the testing data set through various analyses, including detection rate assessment, output distribution analysis, and probability equivalence evaluation for the general faulty class and individual fault types. In order to enhance detection result visualizations, we performed a t-Distributed Stochastic Neighbor Embedding (t-SNE)^55^ on the embeddings from the dense layer when applying the twin unit on the pair time series of the test data sets.

Finally, we compared our findings with results reported in the literature to contextualize our model’s performance. Including the different folds, we trained a total of 131 models during this study, of which individual training durations were recorded. Figure 6 shows a diagram summarizing the four investigation stages conducted in this research.

**Figure 6:**
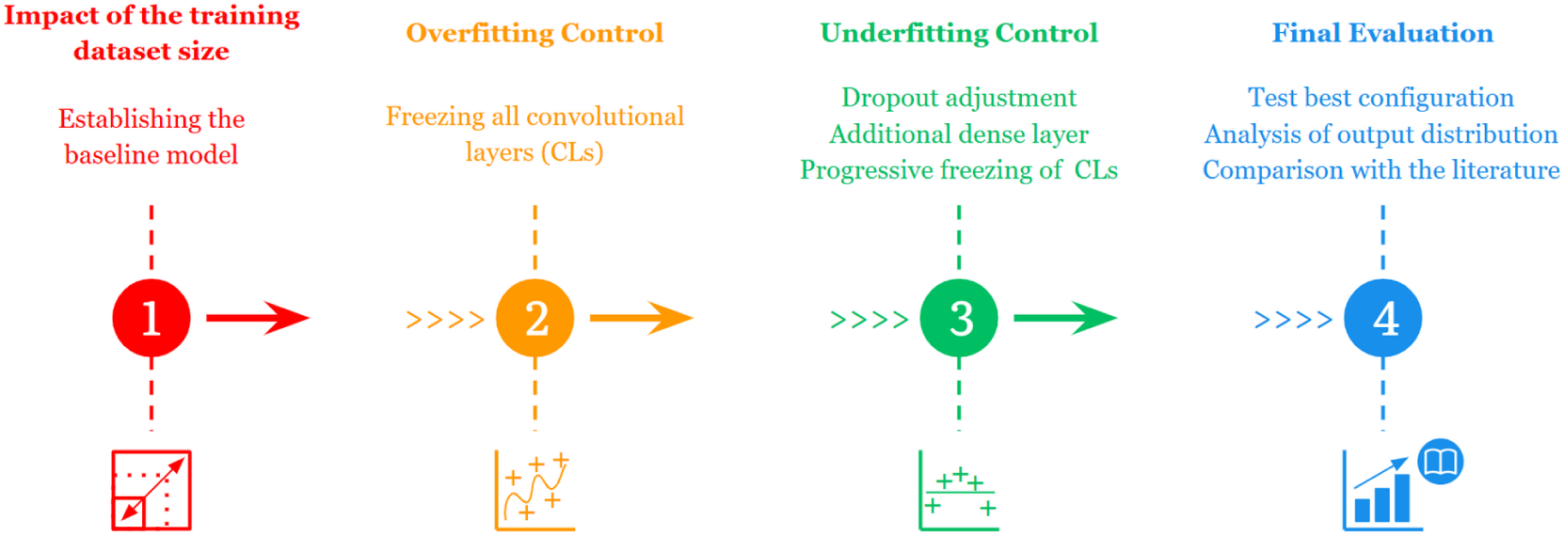
Summary of the investigation stages.

### Model Performance and Training Evaluation Techniques

In this study, the F1-score was chosen as the primary evaluation metric as it is well-suited for classification problems. The F1-score balances precision and recall, which makes it a robust measure for assessing model performance.^56^ Equation 2 presents the expressions that compute precision, recall, and the F1-score. In fault detection, the recall is also referred to as the fault detection rate (FDR),^57^ and we used this metric to evaluate the detection performance of individual faults in the testing stage. Additionally, we analyzed the distribution of predicted distances to investigate the model’s capabilities further.

2 $$\{\begin{array}{l} \mathrm{Precision}=\frac{\mathrm{TF}}{\mathrm{TF}+\mathrm{FF}}\\ \mathrm{Recall}=\frac{\mathrm{TF}}{\mathrm{TF}+\mathrm{FN}}\\ F1_{\mathrm{Score}}=2\times \frac{\mathrm{Precision}\times \mathrm{Recall}}{\mathrm{Precision}+\mathrm{Recall}} \end{array}$$

where TF is true faulty, FF is false faulty, and FN is false normal.

Additionally, we recorded the L2 gradient norm for the baseline model and all subsequent trained cases to monitor training stability and convergence. Equation 3 provides the expression used to compute the L2 gradient norm, which indicates the optimization behavior throughout training.

3 $$L_{2,\mathrm{norm}}=\sqrt{\overset{N}{\underset{i}{\sum }}{\mathrm{gradient}}_{i}^{2}}$$

Since the predicted distance is not bound to a closed range, we applied a modified sigmoid function, as shown in eq 4, to the model’s predictions to better interpret the predicted distance distribution around the margin. This transformation sets the margin to 0.5 and limits the scale between 0 and 1. Furthermore, we used a reliability diagram^58,59^ to assess whether the sigmoid-transformed distance output could be interpreted probabilistically using the calibration_curve function from Scikit-learn setting it to 10 bins.

4 $$\mathrm{sigmoid}\left(D\right)=\frac{1}{1+e^{\left(m-D\right)}}$$

where *D* is the predicted distance and *m* is the margin set in the contrastive loss.

## Results and Discussion

This section presents and analyzes the study findings, dividing them into two main parts. In the first subsection, we examine the impact of data set size on model performance, establish a baseline configuration, and refine key training parameters. In the second part, we further assess the final model’s performance, stability, and generalization capabilities, providing a comparative evaluation against existing literature.

### Training Data Set Size, Baseline, and Hyperparameter Investigations

Figure 7 summarizes the results of the initial investigation. It is possible to observe a considerable increase in the resulting model performance until 5,250 samples (7.5% of the total training data set). The training performance steadily increases for cases of 7,000 or more samples until the entire data set is included. However, performance on the validation set fluctuates considerably for intermediate training data set sizes, reproaching training performance for more extensive training data. The observed gap could be related to overfitting but could also be related to a premature triggering of the early stopping callback.

**Figure 7:**
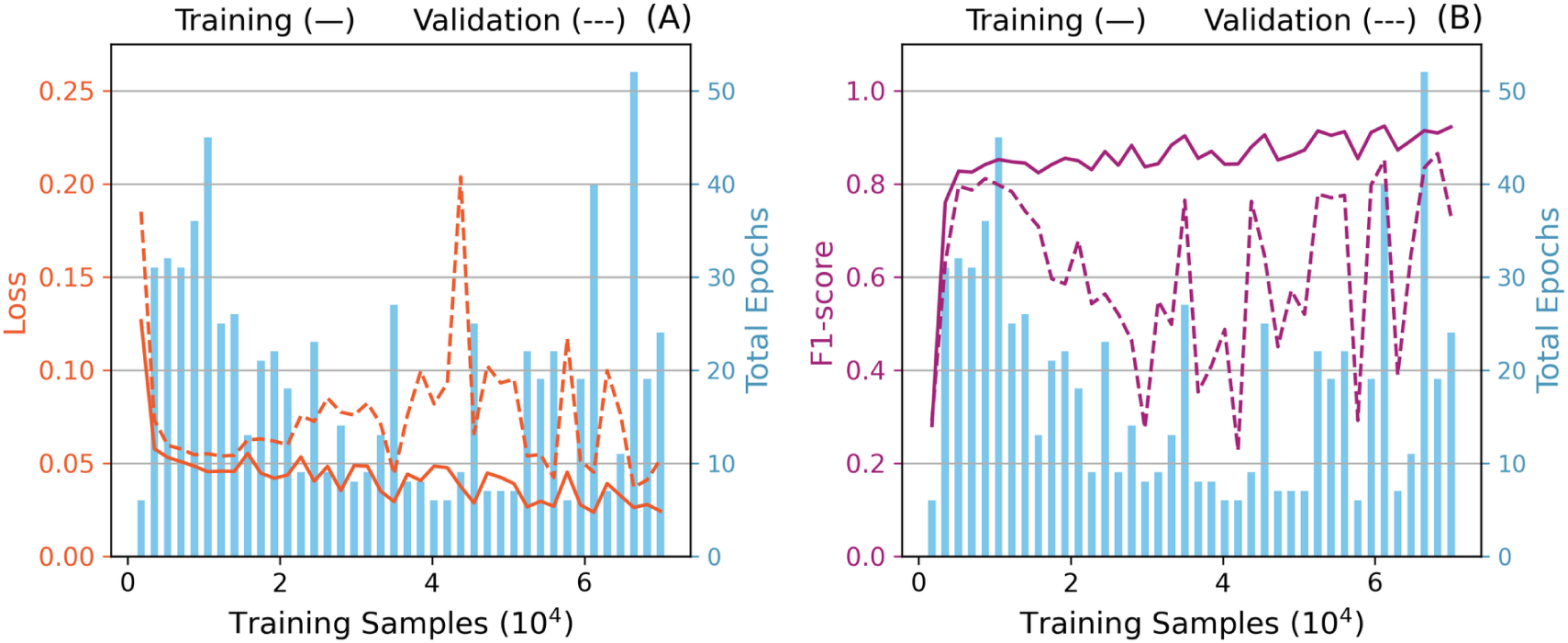
Loss (A) and F1-score (B) learning curves with early stopping and learning rate scheduler patience terms of 5 and 4, respectively.

In fact, most instances of reduced validation performance are associated with a lower number of training epochs. For example, the configuration using 68,250 training samples (97.5% of the data set) yielded the highest validation F1-score (86.50%), with a modest gap of 4.38% compared to the corresponding training F1-score, and completed after 19 epochs. In contrast, the configuration with 42,000 training samples (60% of the data set) resulted in the lowest validation F1-score (22.66%), which was 61.56% lower than its training performance and halted prematurely at just 6 epochs.

From the data presented in Figure 8, it is possible to observe that the increase in the training F1-score is smooth and improves as the training data set size increases. Contrarily, the same score on the validation data has shown an increasing but considerably oscillatory behavior, especially in the initial epochs.

**Figure 8:**
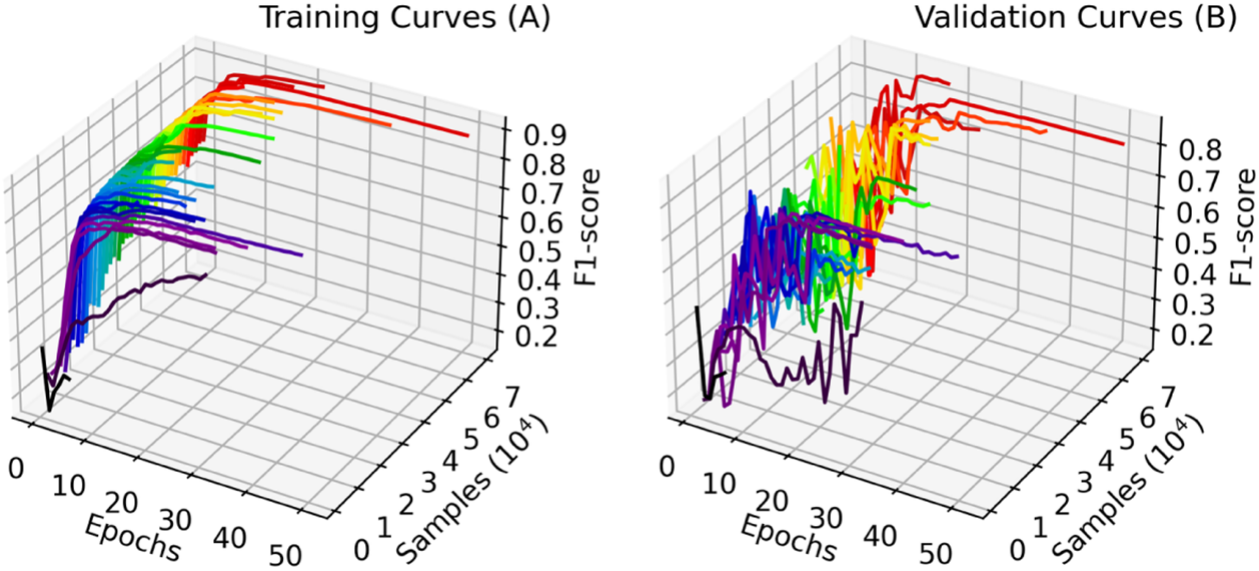
Training (A) and validation (B) learning curves with early stopping and learning rate scheduler patience terms of 5 and 4, respectively.

Such behavior is further evidence of premature early stopping, which indicates that the patience term might be too short. Since cases of better performance stopped between 20 and 40 epochs, we decided to reevaluate the effects of the training set data size with a fixed quantity of epochs equal to 30.

With the learning curve results with the increasing data set size and the fixed quantity of epochs, it was possible to build Figure 9. Similarly to the previous configuration, there is a spike in performance up to 5,250 samples. However, the training performance increased and became more stable for cases with more data.

**Figure 9:**
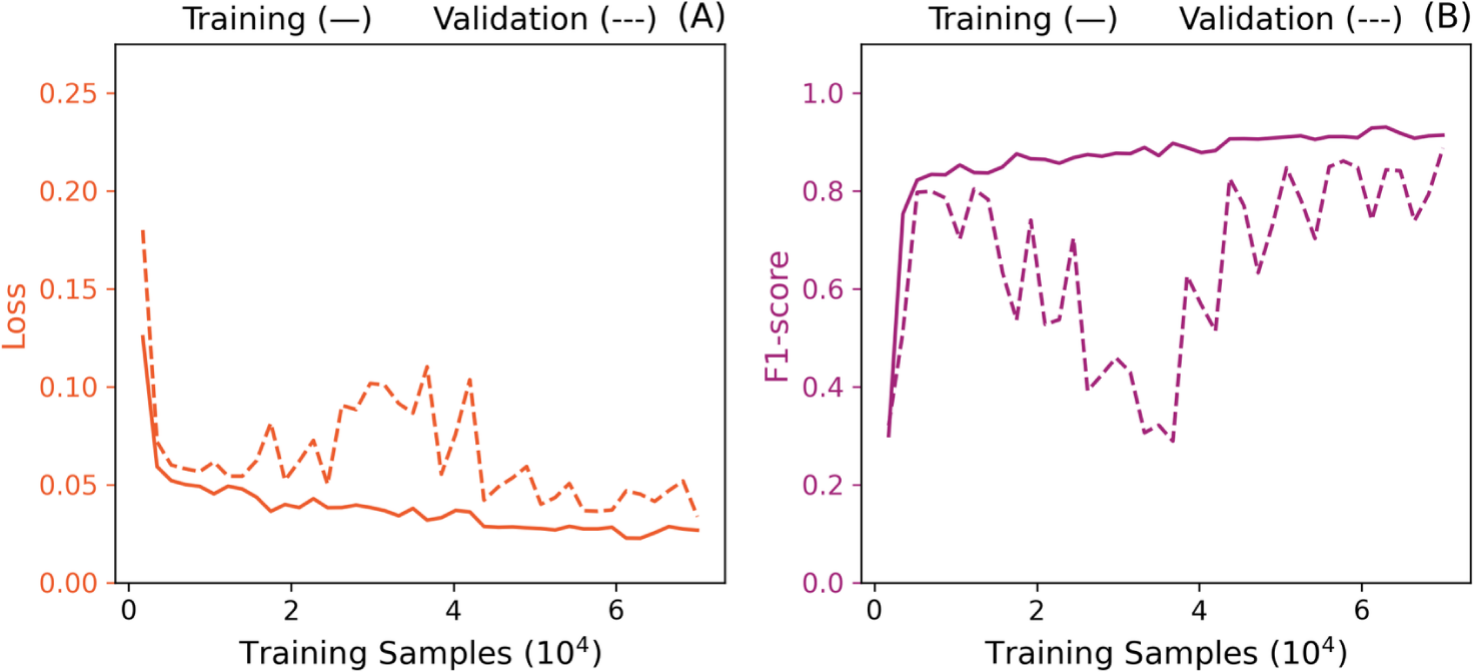
Loss (A) and F1-score (B) learning curves with 30 epochs and a learning rate scheduler patience term of 4.

Although significant gaps for intermediate amounts of training data remained, validation performance showed fewer fluctuations. Meanwhile, cases with more than 43750 training samples presented improved results.

Using the largest training data set in the study corresponded to the highest validation F1-score of 88.89%. This case also presented a 2.25% gap from the training performance, which is a considerable improvement when compared to the previous configuration. These results are an indication that the amount of data we select seems to be appropriate for the problem we are trying to solve.

From observation of the individual training curves shown in Figure 10, it is possible to conclude that intermediate cases reached an early stagnation during training. This effect is most likely related to an early triggering of the learning rate scheduler, which indicates a patience term of 4 is too short. Also, most cases have presented a more oscillatory behavior between 10 and 15 epochs. Due to these reasons, we selected patience terms of 10 and 15 for the lr scheduler and the early stopping, respectively, for the following implementations of this study.

**Figure 10:**
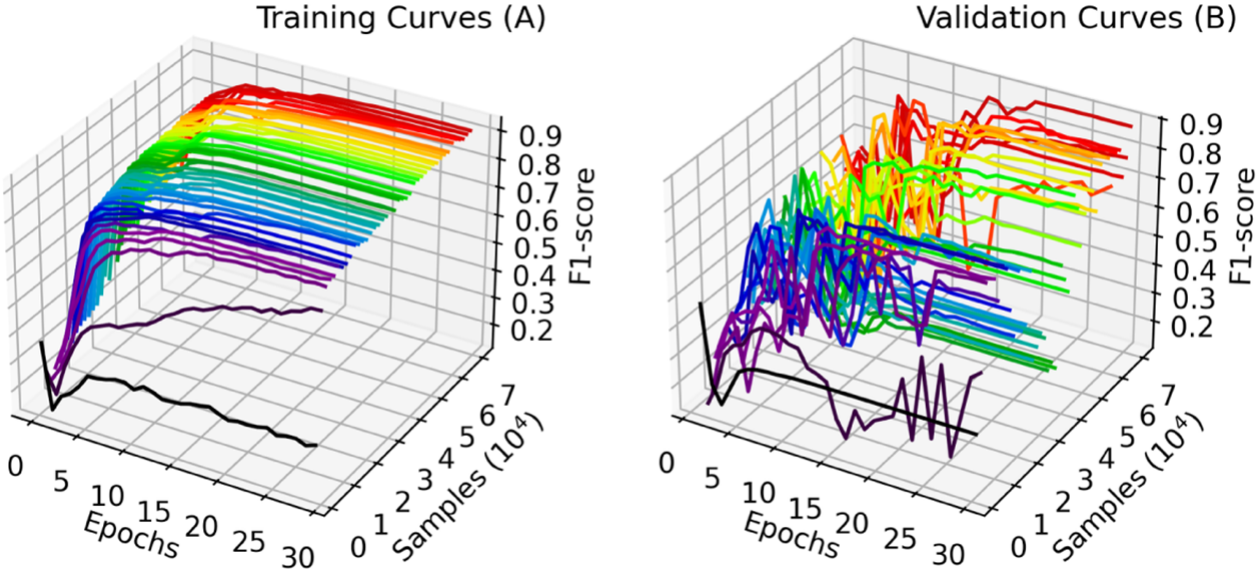
Training (A) and validation (B) learning curves with 30 epochs and a learning rate scheduler patience term of 4.

Having a fixed number of epochs allows us to analyze the impact of the amount of training data samples on the training duration. It is possible to observe a linear relationship between these two factors in Figure 11. In fact, we obtained an *R*2 of 0.9991 after applying linear regression.

**Figure 11:**
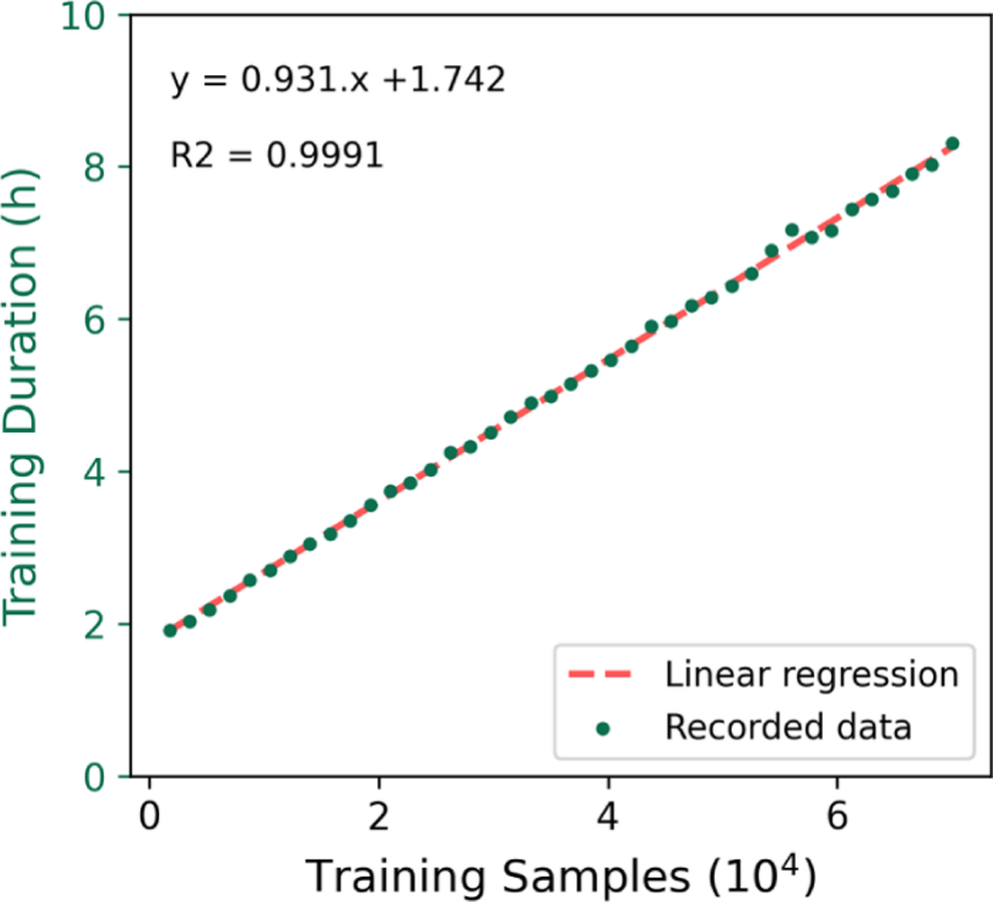
Impact of training data set size on the training duration.

In order to evaluate stability, we performed cross-validation on the baseline model and all other hyperparameter investigations. The baseline implementation reached F1-scores of 93.99 ± 0.93% and 83.85 ± 2.92% for the training and validation data sets, respectively. Besides, the training duration was 11.3 ± 2.7 h with 46.4 ± 11.3 epochs, representing considerable instability. Such behavior can also be observed in Figure 12, as the oscillation on the validation set seems to trigger premature stopping on folds 2, 3, and 4. Additionally, Figure 13 shows the L2 norm of the gradients.

**Figure 12:**
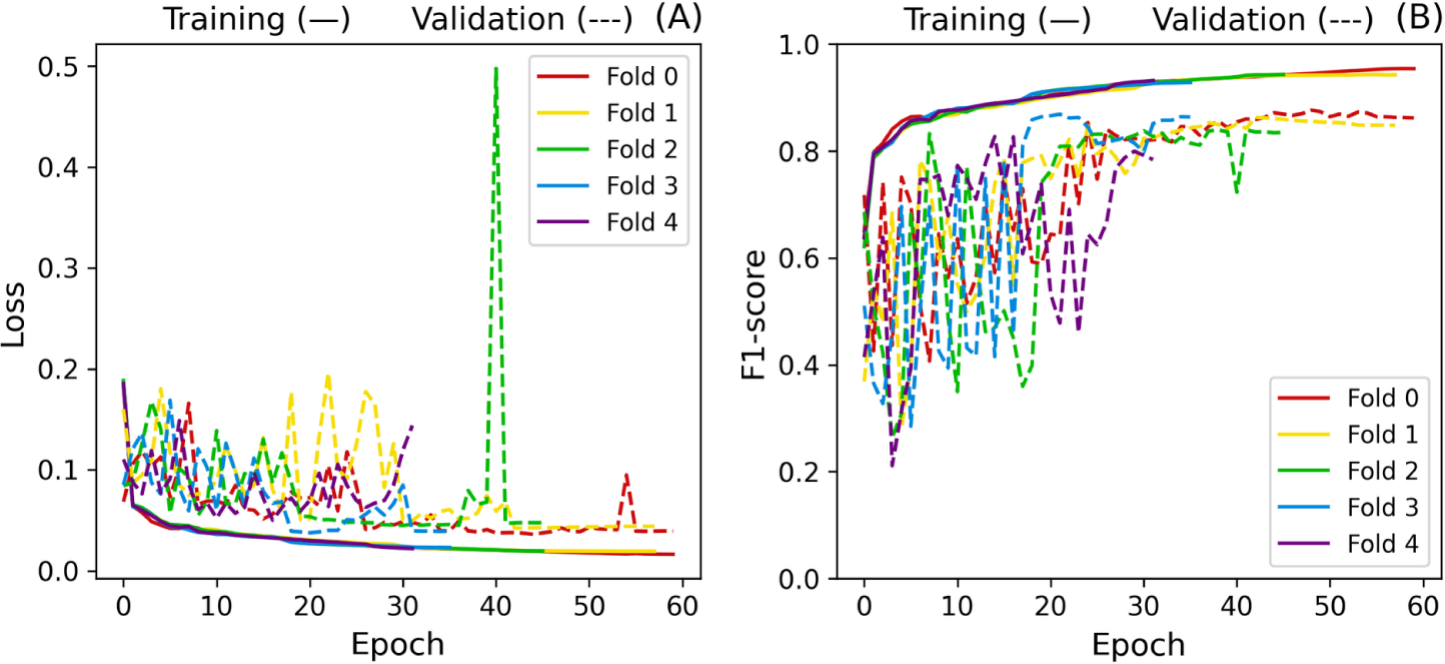
Baseline cross-validation loss (A) and F1-score (B) curves.

**Figure 13:**
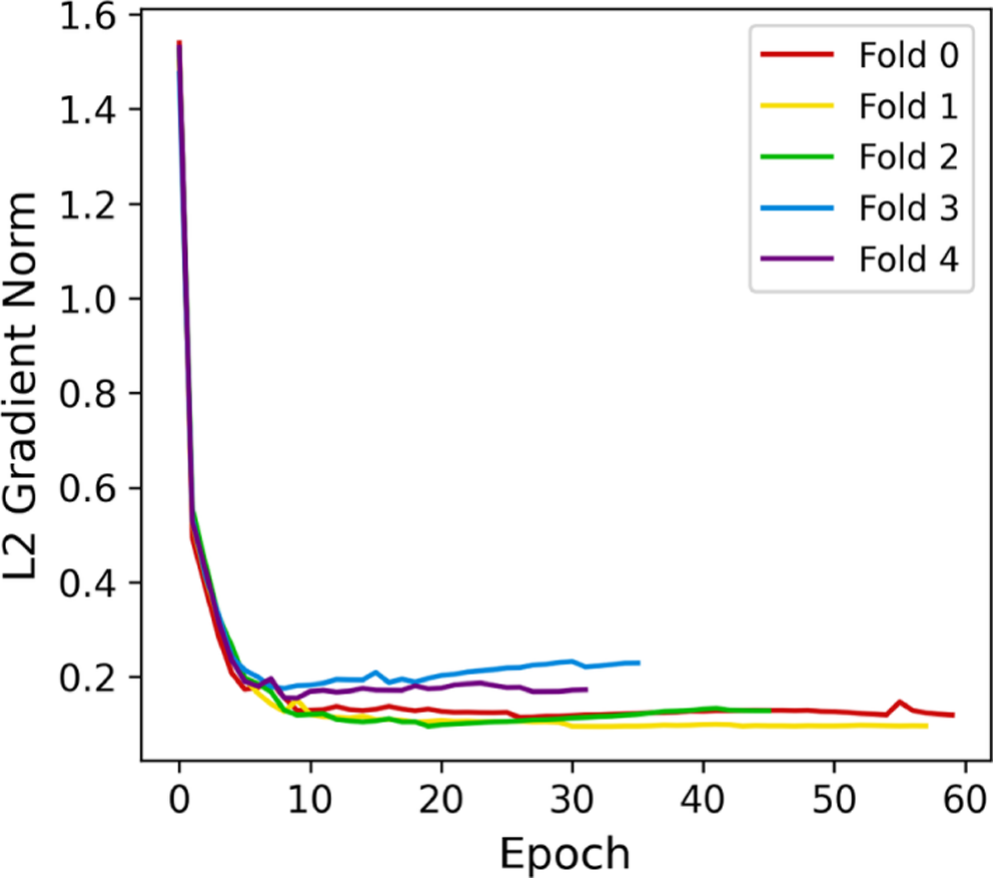
Baseline L2 gradient norm.

Gradients in folds 3 and 4 remained more significant than those in the other folds, indicating more impactful weight updates. A factor that could be contributing to this instability is the amount of trainable parameters in the model (9,597,440), which could also lead to overfitting. Besides, changing the weights from the convolutional layers may shift the model away from the patterns learned from the diagnosis.

Due to the reasons stated above, stage two consisted of freezing all layers before the dense layer of the backbone model. Doing so ensures that the knowledge transferred is not entirely ″forgotten″ while making the extracted features’ processing adaptable.

As expected, this procedure significantly impacted the model’s stability. The cross-validation resulted in 91.41 ± 0.12% of the training F1-score and 90.84 ± 0.18% of the validation F1-score, representing a significant improvement in consistency and generalization. The average difference between training and validation for this metric is 0.57% compared to the 10.14% baseline. Furthermore, the standard deviation was reduced by 0.81% for training and 2.76% for validation, as shown in Figure 14.

**Figure 14:**
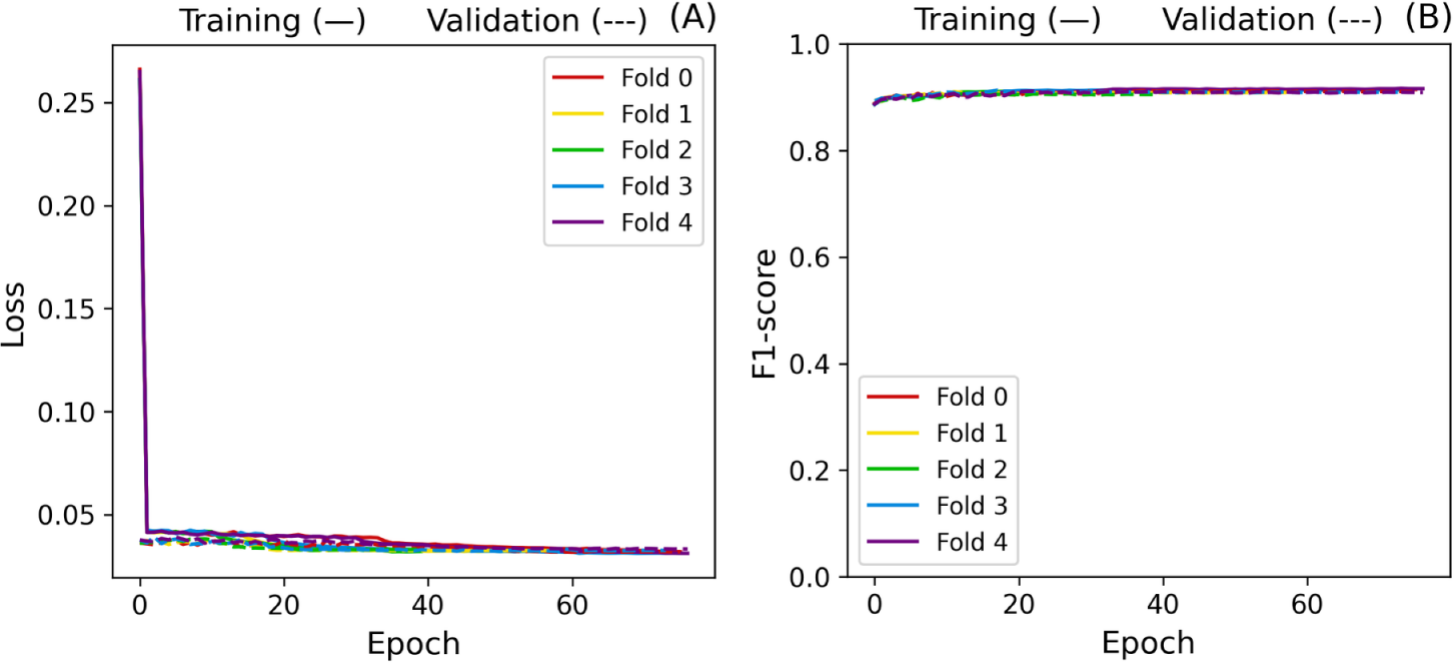
Cross-validation loss (A) and F1-score (B) curves after convolutional layers.

Not only did the model achieve higher performance on its first epoch, but validation metrics persisted smooth throughout the training process. Interesting insights can also be taken from Figure 15. All gradients drop close to 0.22 before increasing again and stabilizing around 0.48. Since all fold starting parameters are the same, this different increasing pace observed could be due to how the optimizer navigates the loss landscape given the fold splits. Nevertheless, they seem to converge to similar final states.

**Figure 15:**
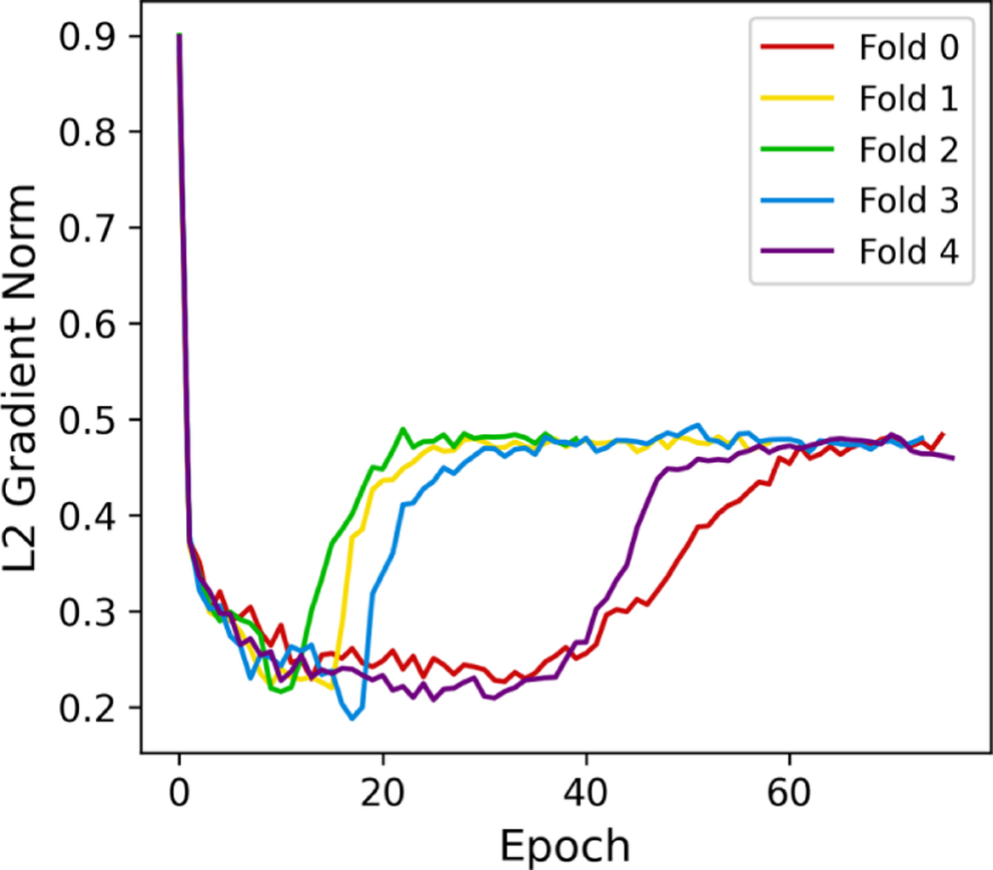
L2 gradient norm after freezing convolutional layers.

Although the number of trainable parameters was 263,168 (9,334,272 less than the baseline model), the training duration was 15.8 ± 3.5 h. This duration increase results from the quantity of epochs executed before training stopped: 65.2 ± 14.2.

Since the model’s performance increases gradually from the first to the last training epoch, we considered the possibility of an over-regularization due to the 50% dropout rate on the dense layer. For this reason, we started stage three by investigating the effects of reducing and removing the dropout effect.

There was no improvement in reducing the dropout effects on the dense layer. Contrarily, removing the dropout layer lowered the training F1-score to 90.95 ± 0.13%, with insignificant changes in validation performance. These results indicate that the 50% dropout rate is not causing over-regularization.

The following hypothesis was that the model did not have enough complexity to process the extracted features further. Consequently, we defined the second part of stage three as evaluating the effects of making the dense part of the model deeper by adding a second dense layer with 512 and 1024 neurons.

In both cases, adding another dense layer resulted in a similar reduction of the F1-score. Besides, the average gap between training and validation for the same metric was 1.32% for 512 neurons and 1.48% for 1024 neurons, which indicates that a second hidden layer might increase the tendency to overfitting. Contrastingly, we observed a significant learning duration reduction in both cases: 6.3 ± 1.2 h stopping at 30.0 ± 6.1 epochs for 512 neurons and 7.1 ± 1.7 h stopping at 33.0 ± 8.6 epochs for 1024 neurons. These results indicate that a lack of complexity on the dense part of the model does not seem to be the limiting factor for prediction enhancement.

Finally, since freezing the entire convolutional portion of the model can limit the model’s flexibility to adapt to the detection problem, the final hyperparameter investigation consisted of progressively freezing the convolutional structure. Figure 16 summarizes the performance for all configurations from stage three of this study.

**Figure 16:**
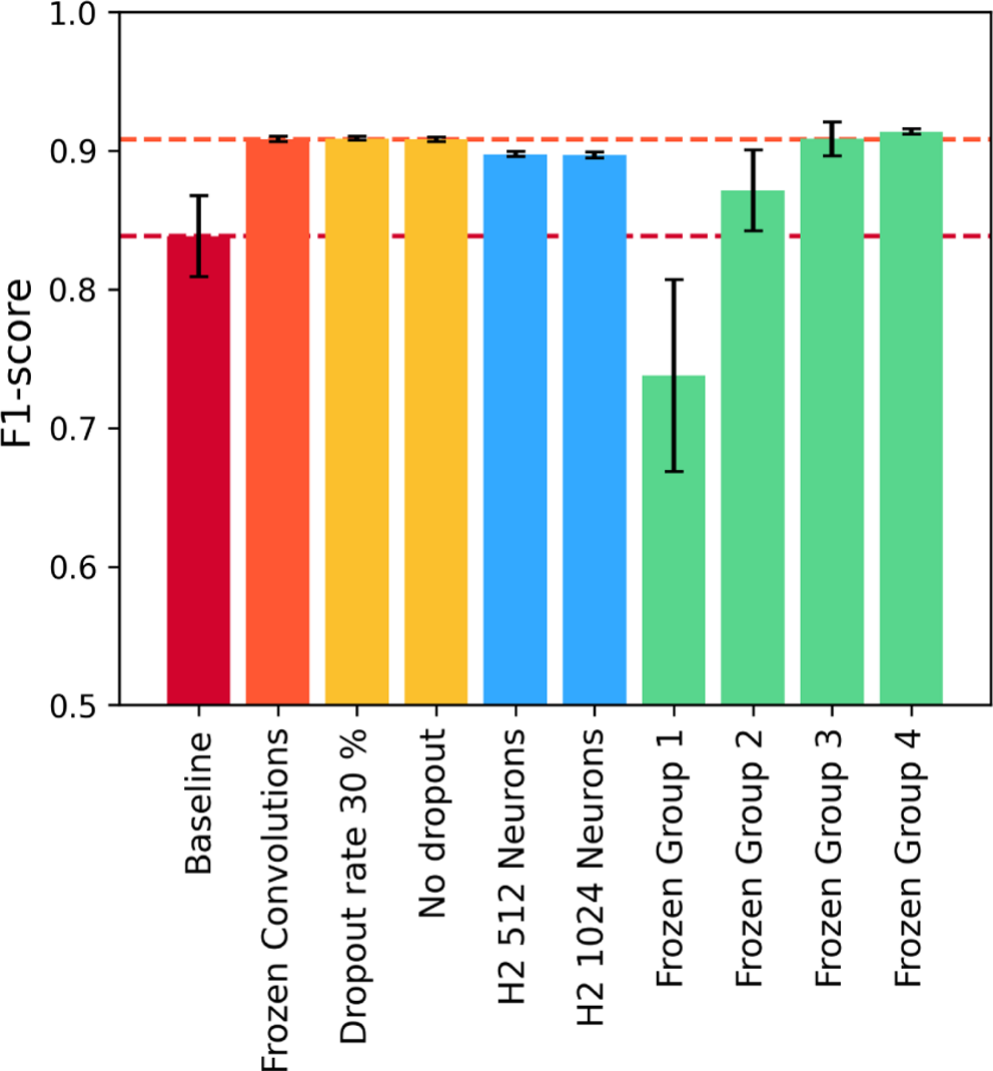
Average cross-validation performance for configurations from stage three.

It is possible to observe that freezing groups one and two, which include fewer layers, increased training performance while lowering scores on validation compared to the baseline. Such behavior indicates overfitting. On the other hand, group three displayed a considerable improvement, with an average of 4.61% F1-score gap between training and validation. This performance is less consistent than the results for freezing all convolutional layers, as freezing group three resulted in a more significant gap between the training and testing performance and larger standard deviations. Given the amount of charts, all training curves and L2 gradient norms for configurations from stage three can be found in the Supporting Information.

The model obtained from freezing group four presented this study’s highest cross-validation average performance. It presented training and validation F1-scores of 92.48 ± 0.49% and 91.38 ± 0.20%, respectively, implicating an average gap of 1.10%. Although these variation ranges are wider than freezing all convolutions, we chose this as the best configuration in the study because they are lower than 1.50% with improved average performance. Besides, training achieved convergence in fewer iterations: 44.8 ± 4.9 epochs. Consequently, the training duration was 5.1 h faster on average, taking 10.7 ± 1.1 h, even with 853,760 trainable parameters out of 9,602,562.

In the context of stability, although the model presented fluctuations when evaluating the validation data set, it is possible to observe in Figure 17 that they are more prominent in the initial learning stages. Additionally, although the increase in the validation loss triggered early stopping, the F1-score of the last epoch was consistently the highest. This phenomenon indicates that the loss function does not directly represent our primary classification metric. This difference is expected since the contrastive loss is distance-based, while the F1-score is classification-based. Due to this trend, we kept the last state of the model for the last part of the study.

**Figure 17:**
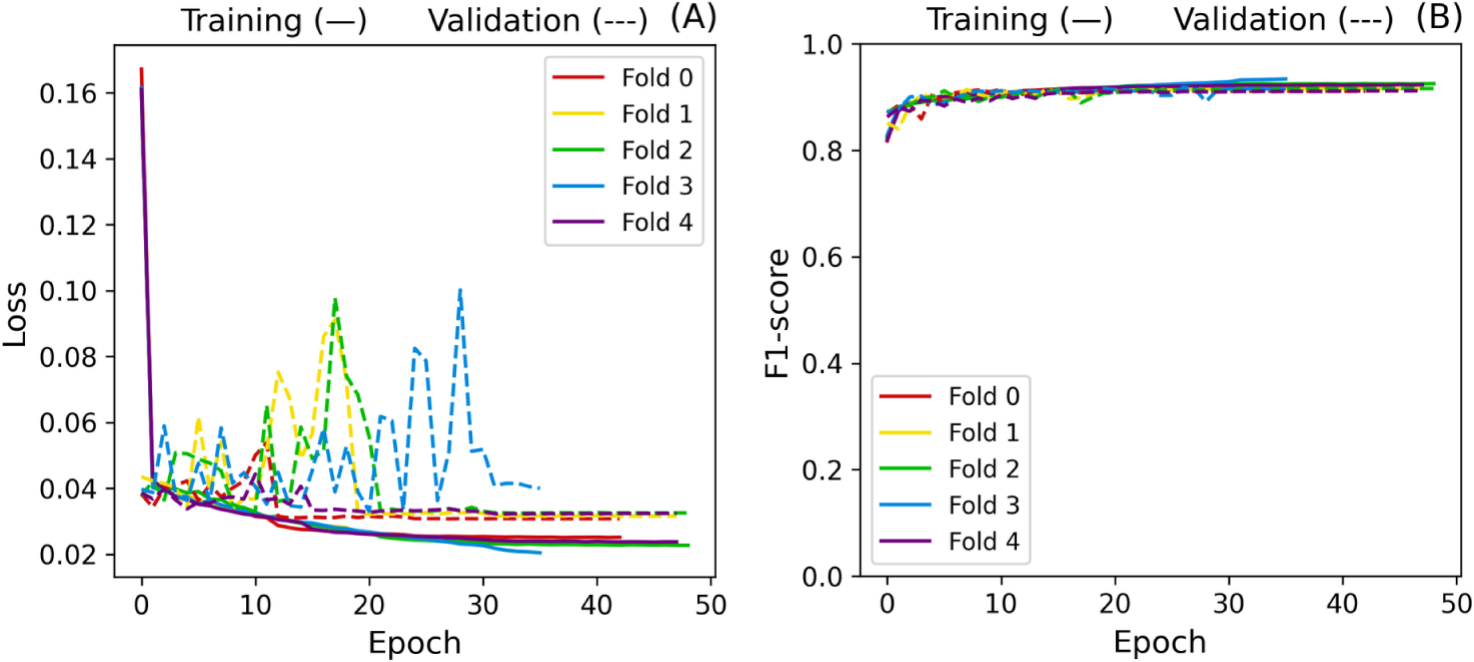
Cross-validation loss (A) and F1-score (B) curves after freezing Group 4.

Regarding the L2 gradient norm shown in Figure 18, all folds presented an overlapping steady decline behavior. However, folds 0 and 4 became stable at slightly higher values than the others, which is insignificant compared with previous cases.

**Figure 18:**
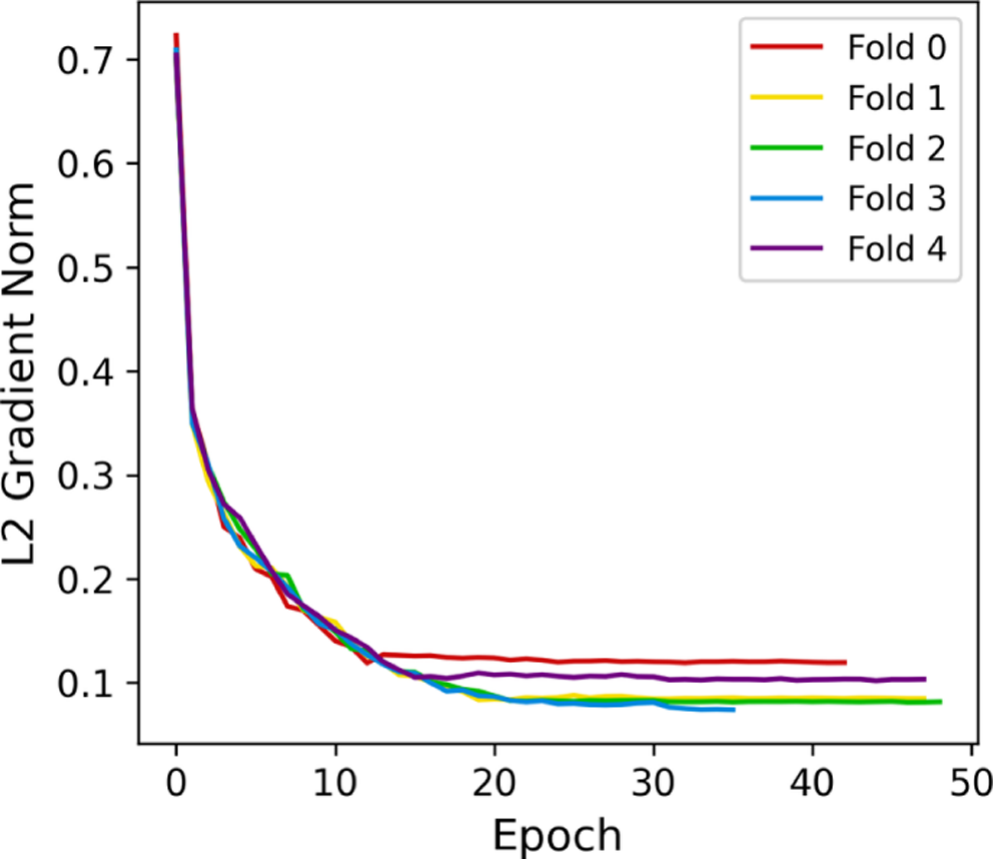
L2 gradient norm after freezing for Group 4.

Since the last convolutional layers are responsible for extracting the most abstract information related to the investigated problem, it is coherent that keeping these layers trainable was beneficial to the learning process. Due to the improved performance and stability observed, we selected freezing of the layers in group four as the best configuration within the scope of this study. Table 2 summarizes the results of all of the parameters that we investigated in our research.

**Table 2:** Summary of the Investigated Hyperparameter Results

	F1-score (%)			
Case	Training	Validation	Epochs	Duration (h)
Baseline	93.99 ± 0.93	83.85 ± 2.92	46.4 ± 11.3	11.3 ± 2.7
Frozen Convolutions	91.41 ± 0.12	90.84 ± 0.18	65.2 ± 14.2	15.8 ± 3.5
30% dropout	91.31 ± 0.07	90.89 ± 0.15	65.2 ± 13.2	13.5 ± 2.7
No dropout	90.95 ± 0.13	90.82 ± 0.15	62.6 ± 13.0	13.1 ± 2.6
H2 512 Neurons	91.07 ± 0.10	89.76 ± 0.18	30.0 ± 6.1	6.3 ± 1.2
H2 1024 Neurons	91.17 ± 0.09	89.69 ± 0.21	33.0 ± 8.6	7.1 ± 1.7
Frozen Group 1	93.91 ± 0.92	73.77 ± 6.95	28.0 ± 0.9	6.9 ± 1.5
Frozen Group 2	95.29 ± 0.49	87.13 ± 2.91	31 ± 3.7	7.6 ± 1.0
Frozen Group 3	95.46 ± 0.22	90.85 ± 1.25	28.2 ± 3.4	6.9 ± 0.8
Frozen Group 4	92.48 ± 0.49	91.38 ± 0.20	44.8 ± 4.9	10.7 ± 1.1

### Analysis of the Best Configuration

We trained the model with the best configuration from scratch using the holdout method and the initial training and validation split for testing evaluation. Figures 19 and 20 show that the holdout implementation yielded the same training behavior as that observed in the cross-validation investigation. The training reached 44 epochs before early stopping, taking 8.8 h to finish, with training and validation F1-scores of 92.74% and 91.57%, respectively.

**Figure 19:**
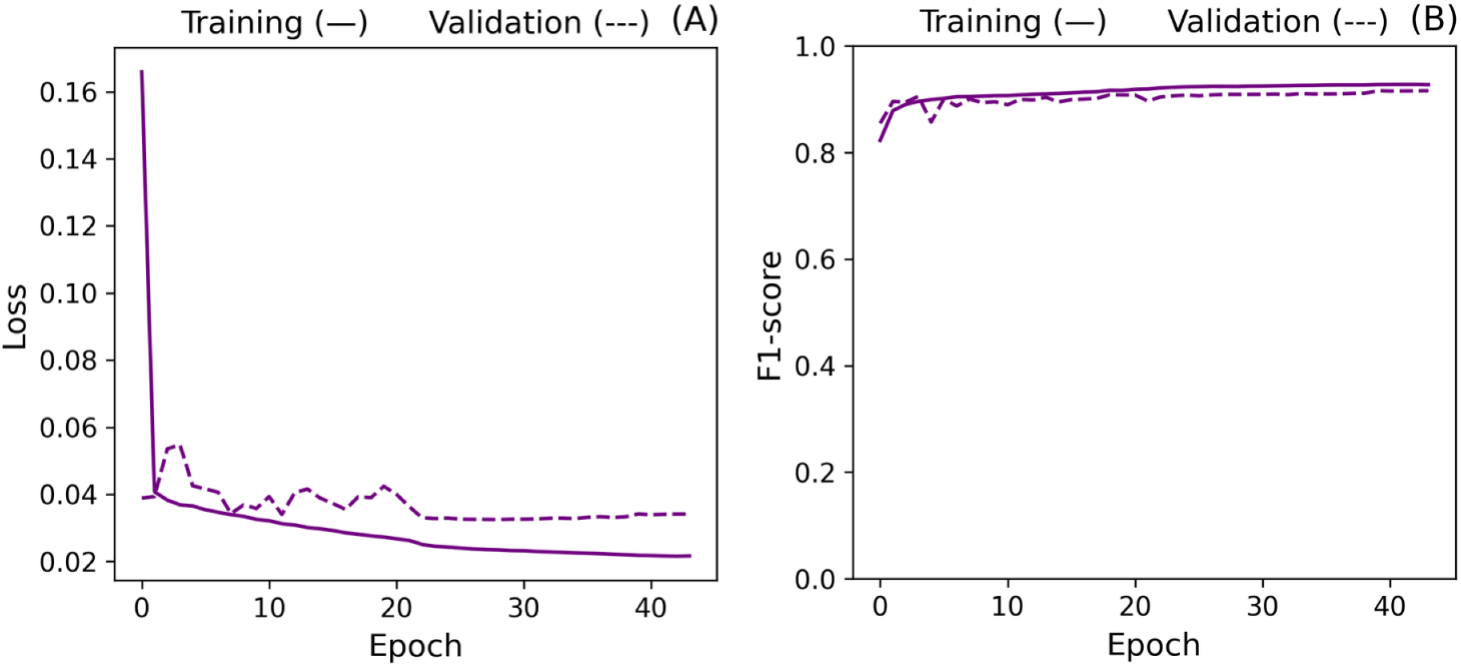
Best model’s holdout loss (A) and F1-score (B) curves.

**Figure 20:**
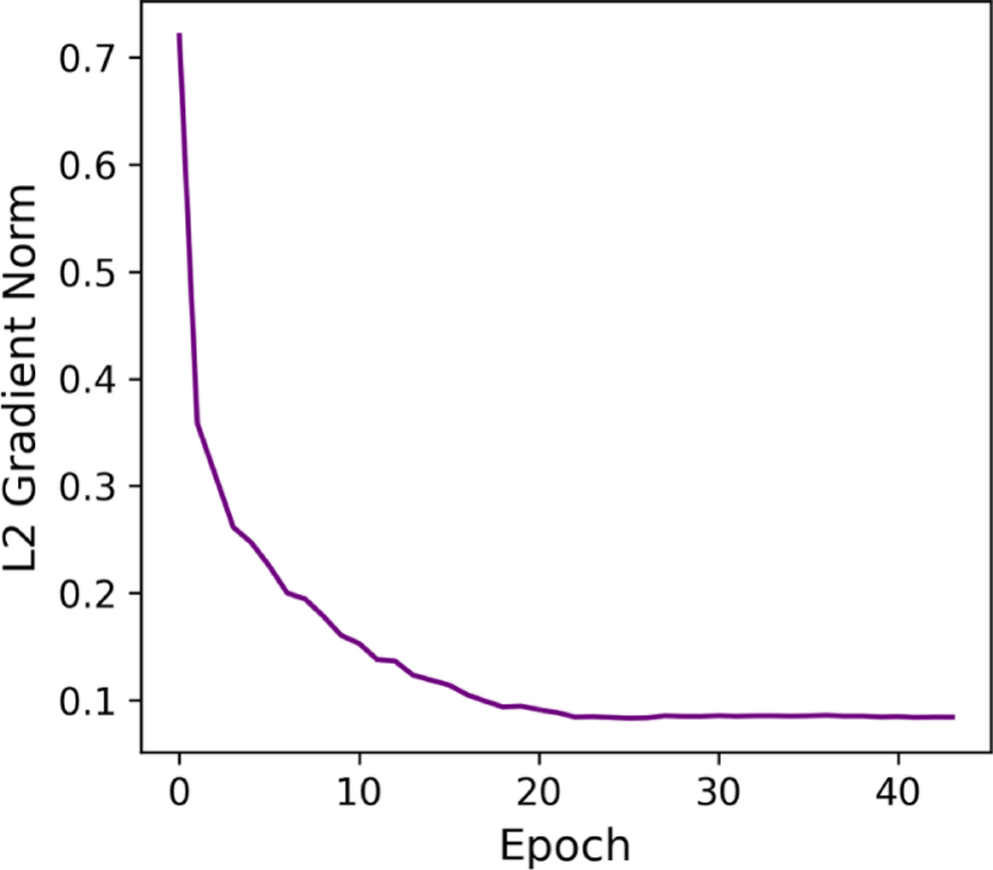
Best model’s holdout L2 gradient norm.

In terms of deployment feasibility, the average inference time of the best-performing model was 0.011 s per test sample. This is substantially faster than the data set’s sampling interval of 3 min, indicating that the model is capable of operating in real time with considerable margin. This suggests a strong potential for industrial implementation, as the model can deliver timely predictions with minimal computational overhead once trained. Performance-wise, the model achieved an F1 score of 91.41% and a contrastive loss of 0.072, which is close to the validation, indicating that no significant overfitting effect occurred.

Analyzing the distribution of distances predicted by the model is important for a better understanding of its behavior. Figure 21 shows a strongly skewed distribution toward zero for cases of normal behavior, with an average predicted distance of 0.11 and a standard deviation of 0.16. This distribution indicates that the model was able to learn new patterns that were not directly present in the transferred knowledge. On the other hand, in the case of faulty behavior, the distance distribution is wide, with an average of 9.08 ± 6.01, which is expected as multiple types of faults can result in different embedding representations. Nevertheless, the majority of faulty cases stay above the margin.

**Figure 21:**
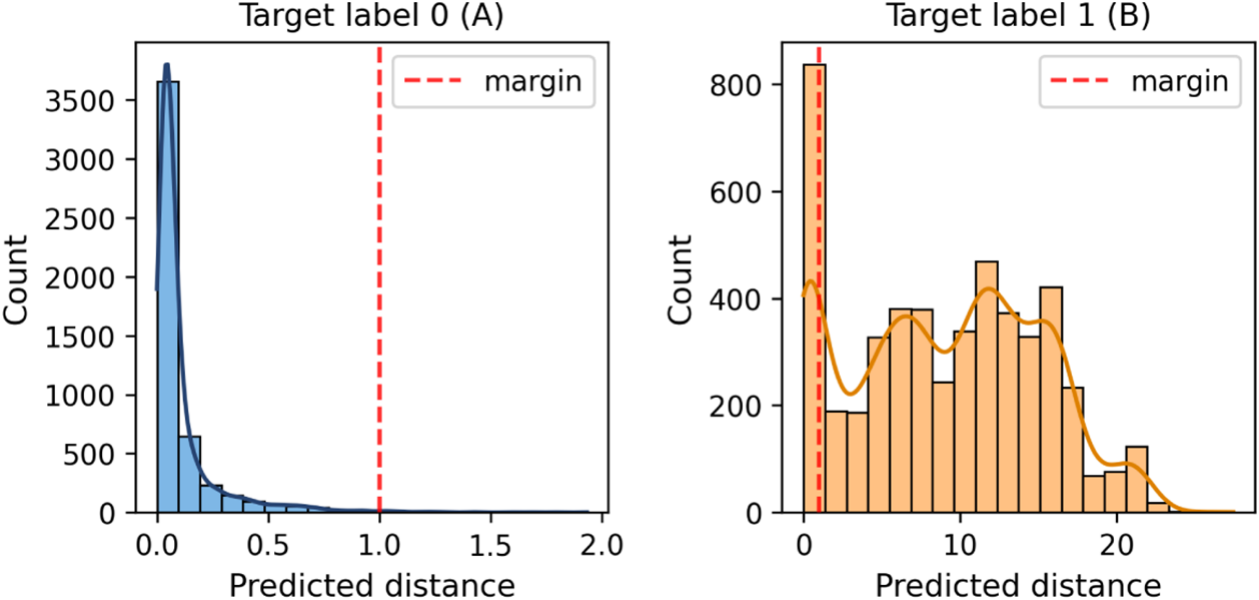
Distribution of predicted distances of test normal (A) and faulty (B) cases.

There is no limit to the model’s predicted distances, which could result in misleading interpretations of the distribution charts. In contrast, the sigmoid function smoothly sets the values between zero and one. Consequently, it is possible to better represent the classification distribution using eq 4. Figure 22 shows that most faulty cases are positioned far from the margin, except for a subset of misclassifications.

**Figure 22:**
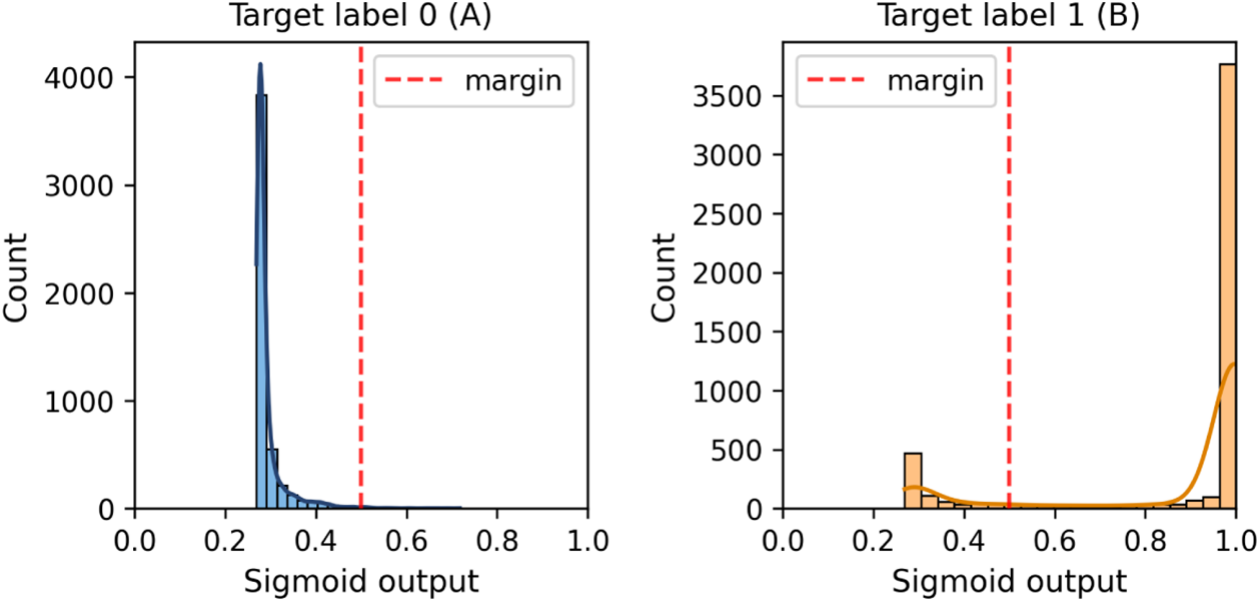
Sigmoid output of the test normal (A) and faulty (B) cases.

The reliability diagram from the transformed values is shown in Figure 23. As the curve indicates poor calibration, we conclude that this output should not be treated as a true probability. Instead, it functions as a normalized similarity score. Importantly, the model still performs reliably on normal samples, as reflected in the classification metrics and distribution analysis.

**Figure 23:**
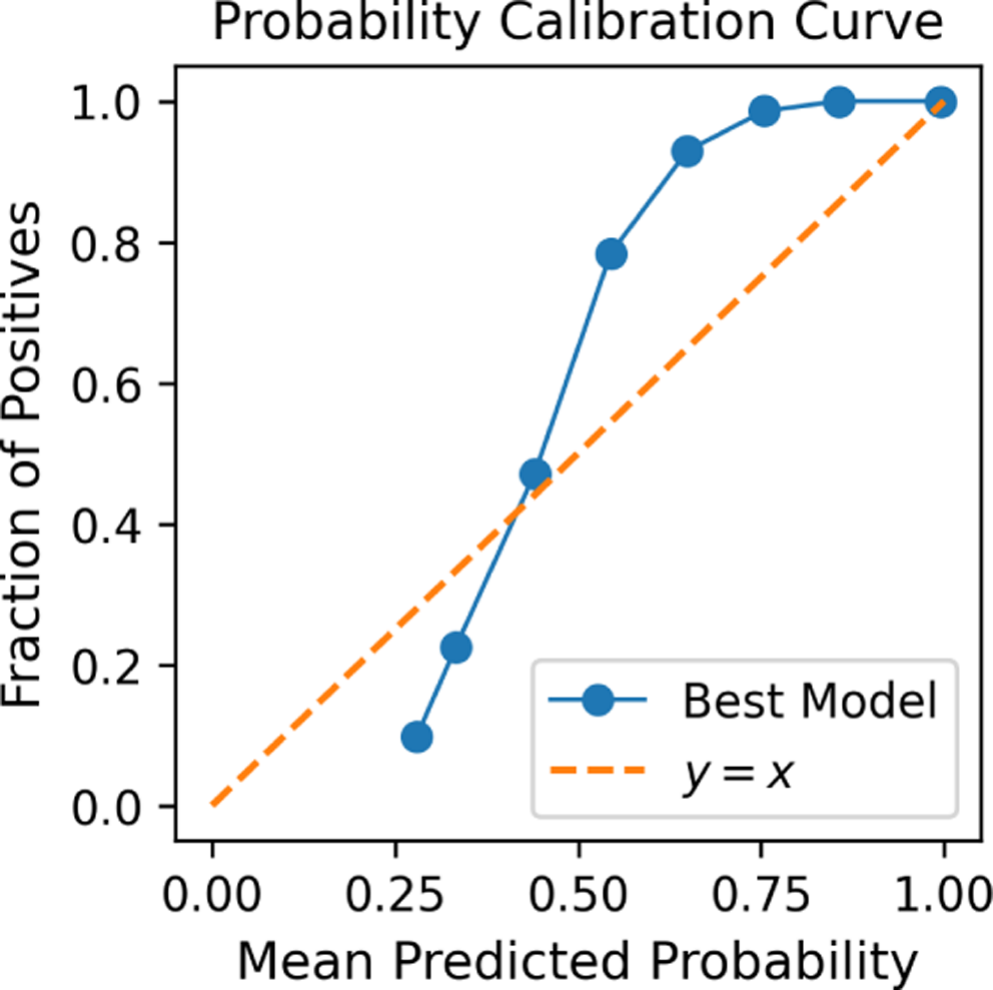
Probability calibration curve of our best model.

Although we present a probability calibration curve, our primary aim was not to achieve a calibrated probability output but rather to assess whether the transformed scores behaved like the probabilities. The observed curve indicates that the model’s output deviates from ideal calibration. While postprocessing techniques such as temperature scaling or threshold adjustment could potentially improve calibration, these were not pursued in this study, as our objective was to analyze the inherent behavior of the model’s transformed output rather than to optimize it for calibrated probabilistic predictions.

Since we have access to the specific fault label of each sample, we analyzed each fault to better understand the detection predictions. It is important to mention that these results are still based on the normal versus faulty predictions from the model and not to be mistaken with fault diagnosis modeling. Table 3 summarizes the detection rate and statistical information on predictions.

**Table 3:** Individual Fault Results and Average Model Outputs

Fault Case	FDR	Average Distance	Average Sigmoid Output
Fault 1	1.0000	11.94 ± 1.64	1.00 ± 0.00
Fault 2	1.0000	15.55 ± 1.36	1.00 ± 0.00
Fault 3	0.3911	0.93 ± 0.82	0.48 ± 0.18
Fault 4	1.0000	6.87 ± 1.10	0.99 ± 0.01
Fault 5	1.0000	6.92 ± 2.30	0.99 ± 0.04
Fault 6	1.0000	20.08 ± 1.98	1.00 ± 0.00
Fault 7	1.0000	16.96 ± 1.54	1.00 ± 0.00
Fault 8	0.9959	10.92 ± 3.23	0.99 ± 0.05
Fault 9	0.1399	0.45 ± 0.70	0.37 ± 0.15
Fault 10	0.8385	4.95 ± 3.81	0.82 ± 0.24
Fault 11	0.9958	9.08 ± 3.53	0.98 ± 0.07
Fault 12	0.9959	10.74 ± 2.88	0.99 ± 0.05
Fault 13	0.9562	10.80 ± 3.07	0.97 ± 0.14
Fault 14	1.0000	11.62 ± 0.96	1.00 ± 0.00
Fault 15	0.0075	0.14 ± 0.20	0.30 ± 0.04
Fault 16	0.8618	6.98 ± 5.68	0.85 ± 0.23
Fault 17	0.9788	14.33 ± 3.63	0.98 ± 0.10
Fault 18	0.9014	12.46 ± 4.76	0.93 ± 0.21
Fault 19	0.9919	4.73 ± 1.45	0.95 ± 0.09
Fault 20	0.9291	5.89 ± 2.54	0.93 ± 0.18

Faults 3, 9, 10, 15, and 16 were the cases with the worst performances, which is consistent with what we observed in our previous diagnosis study. Faults 10 and 16 remained above 80%, while the others were undetectable in more than 50% of cases, with an average predicted distance below the margin. Figure 24 shows how the model’s predictions in the three most critical cases are more skewed toward normality. All faults with an FDR above 90% resulted in distributions of the sigmoid transformation heavily leaning toward 1.0. The complete collection of histograms of distances and sigmoid transformations for each fault can be found in the Supporting Information.

**Figure 24:**
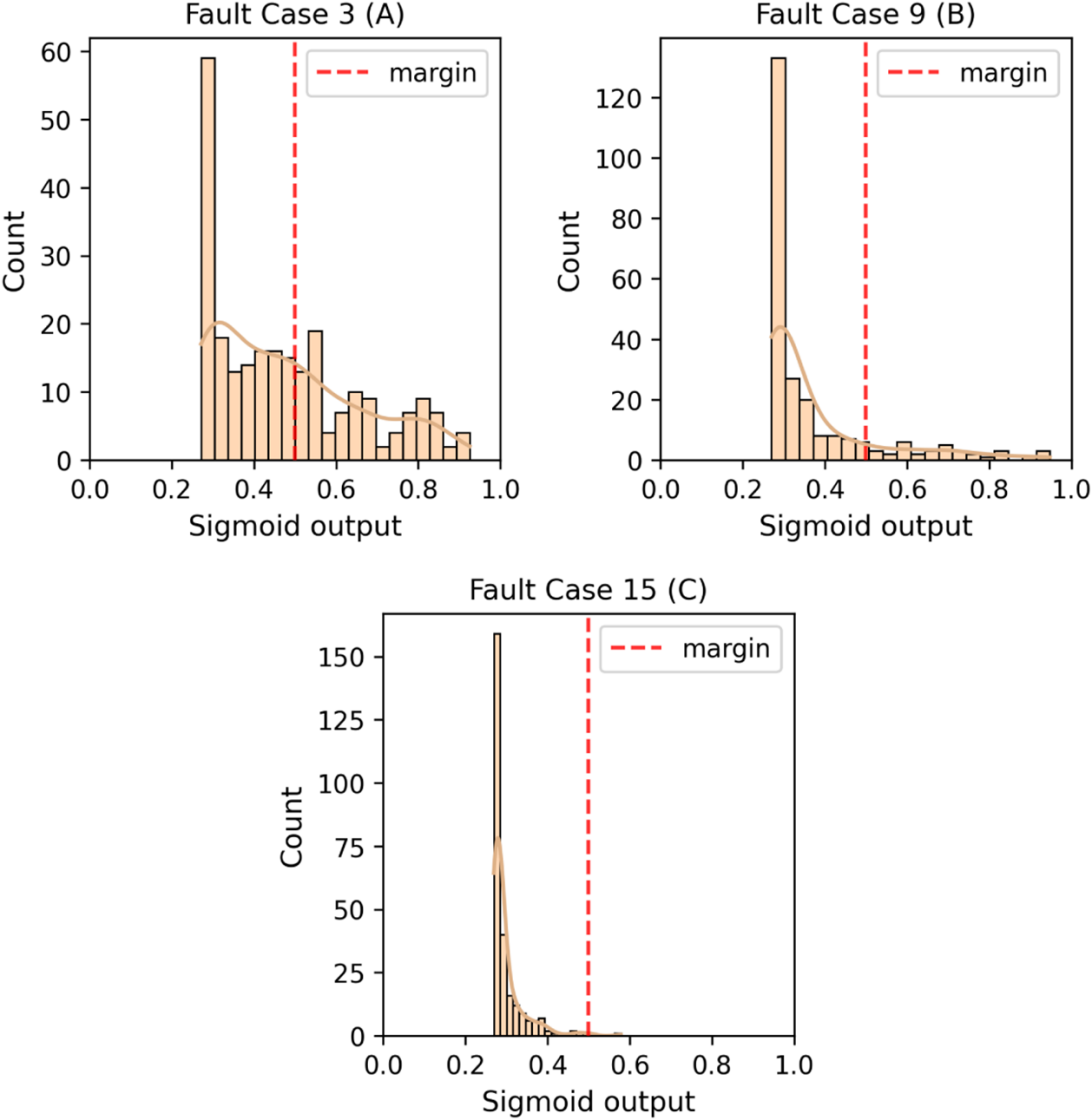
Test sigmoid output of fault cases 3 (A), 9 (B), and 15 (C).

We applied t-SNE to project the embeddings from the dense layer into a lower-dimensional representation to visualize the high-dimensional feature space. Class zero represents fault-free samples, while each of the other classes corresponds to a fault with the same number as the class.

It can be observed in Figure 25 that normal cases occupy a broad region of the plot, whereas certain fault classes form more compact clusters. To improve the analysis of the relationship between detection performance and the distribution of embeddings, we separated the data from t-SNE into two visualizations.

**Figure 25:**
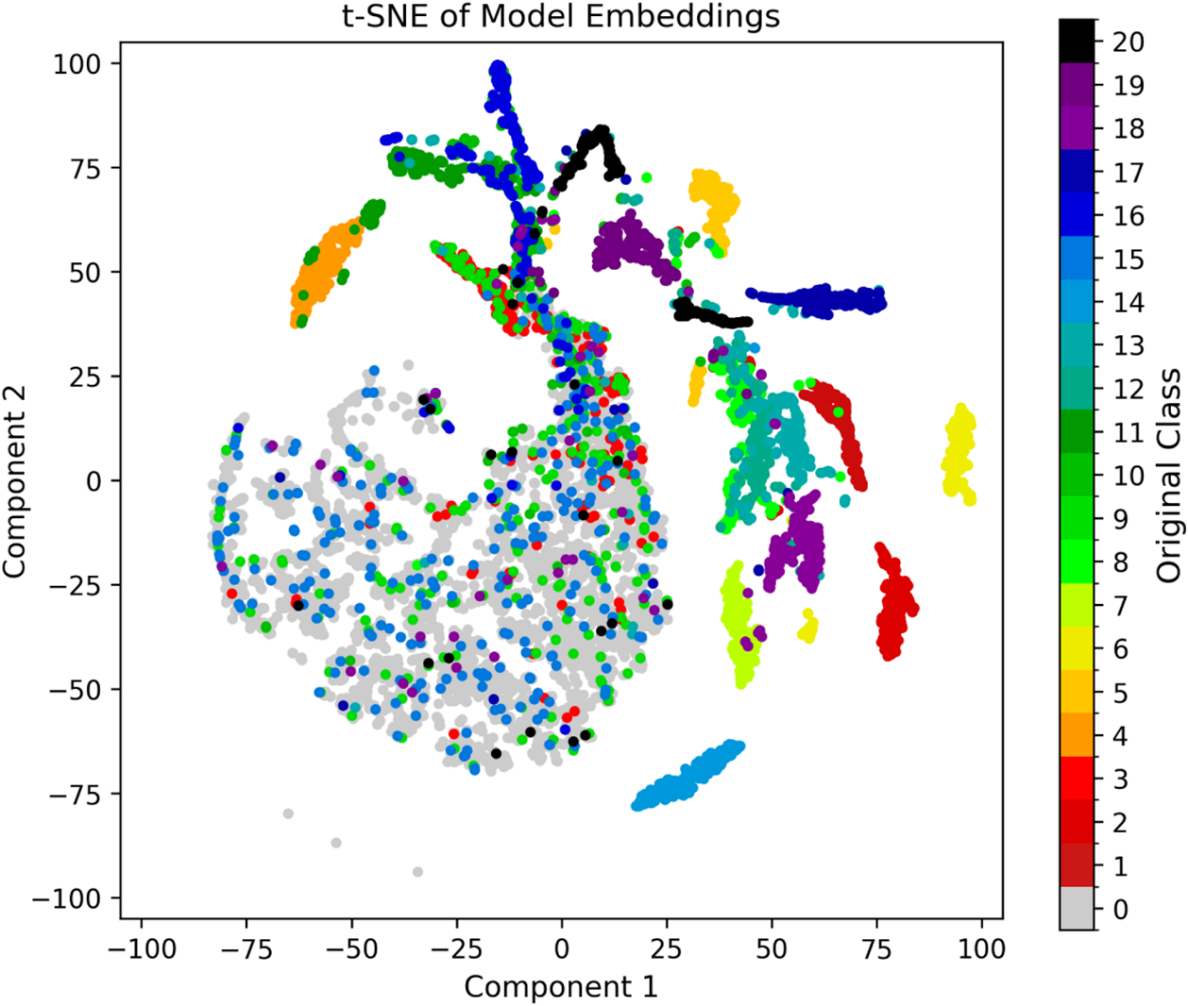
t-SNE of the embedding of the pair series from the test data set.

Figure 26 presents the normal cases alongside fault classes according to their detection rates for better visualization. In Figure 26A, we observe that faults 10 and 16 seem to have individual clusters with partial overlap with the normal case samples. However, cases 3, 9, and 15 are dispersed and overlap substantially with normal behavior, indicating that the model was unable to detect patterns in these cases.

**Figure 26:**
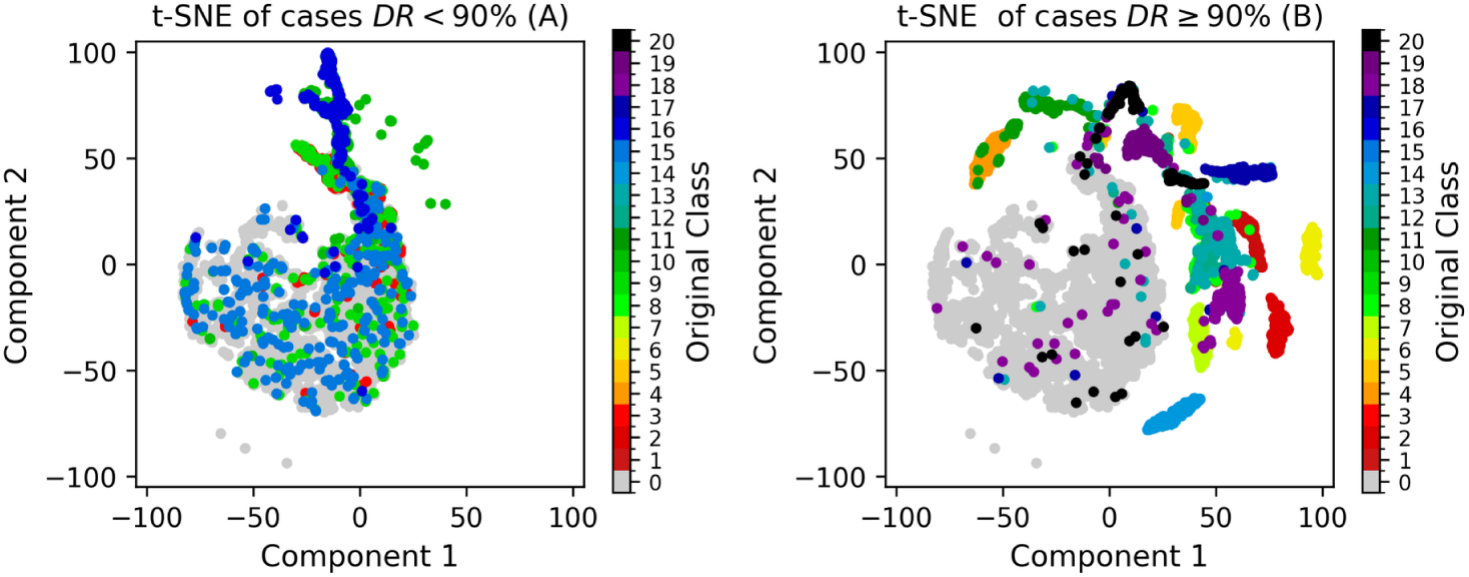
t-SNE of faults from the test data set separated by the detection rate (DR) lesser than 90% (A) and greater or equal to 90% (B).

From analyzing Figure 26B, it is possible to observe that, in addition to being significantly separate from normal behavior, some cases show little overlap with other faulty conditions. This result indicates that the model could adapt characteristics from the diagnosis model to the detection task as our contrastive loss setup does not focus on optimizing discrimination between faulty classes.

To evaluate the effectiveness of our model, we compared its detection rate with three recent studies of the TEP. Ma et al. (2024)^60^ investigated an autoencoder architecture with a multiblock orthogonal long short-term memory backbone (MOLA). Their model used 30 input variables divided between 4 processing blocks according to specific parts of the chemical plant. Similar to our study, they included the original 20 fault cases.

Alternatively, Hu et al. (2025)^61^ developed joint time-serial variation analysis (JTSVA), which integrates three sequential data feature extraction techniques, aiming to achieve enhanced discrimination of faults. This study explored 17 of the original faults in addition to the most recent fault not included in our study.

Dong et al. (2025)^62^ proposed a hybrid model focusing on distinguishing anomalous behavior from nonstationary trends. This method uses slow feature analysis with the local outlier factor algorithm to improve model detection through dynamic updates of fault-sensitive variables. Their study considered all of the TEP faults.

Table 4 summarizes the FDRs reported in the studies mentioned alongside the results from our best model for the 20 original TEP faults. Our model achieved the highest detection performance for 65% of the faults. We have identified faults 3, 9, 10, 15, and 16 as the hardest to detect, which is also true for most references. Notably, Ma et al. (2024)^60^ reached higher performance in these cases at the expense of lowering FDRs of easier scenarios. Finally, in cases 12 and 18, our model is on par with the best scores of the references, staying within 0.3%.

**Table 4:** FDR Comparison Between Our Best Model and State-of-the-Art Works from the Literature^d^

Fault Case	Our Model	ref.1^a^	ref.2^b^	ref.3^c^
Fault 1	**1.0000**	0.9983	**1.0000**	0.998
Fault 2	**1.0000**	0.9767	0.9838	0.984
Fault 3	0.3911	**0.9567**	N/A	0.688
Fault 4	**1.0000**	**1.0000**	**1.0000**	0.998
Fault 5	**1.0000**	0.2633	**1.0000**	0.249
Fault 6	**1.0000**	**1.0000**	**1.0000**	**1.000**
Fault 7	**1.0000**	**1.0000**	**1.0000**	**1.000**
Fault 8	**0.9959**	0.9433	0.9813	0.981
Fault 9	0.1399	**0.8850**	N/A	0.418
Fault 10	0.8385	0.8833	**0.8950**	0.808
Fault 11	**0.9958**	0.9533	0.7888	0.993
Fault 12	0.9959	0.6467	0.9988	**0.996**
Fault 13	**0.9562**	0.8733	0.9525	0.941
Fault 14	**1.0000**	0.9967	**1.0000**	0.780
Fault 15	0.0075	**0.9850**	N/A	0.418
Fault 16	0.8618	0.6450	**0.9263**	0.893
Fault 17	**0.9788**	0.9233	0.9650	0.905
Fault 18	0.9014	0.7633	**0.9038**	0.903
Fault 19	**0.9919**	0.9817	0.9163	0.870
Fault 20	**0.9291**	0.7933	0.9100	0.905
Average FDR	0.8492	**0.8734**	N/A	0.836
Average excluding 3, 9, and 15	**0.9674**	0.8613	0.9542	0.894

a Ma et al. (2024).^60^

b Hu et al. (2025).^61^

c Dong et al. (2025).^62^

d The bold values indicate the highest performance per type of faults (row).

Our model demonstrates strong fault detection capabilities, achieving an average FDR of 0.8492 across all 20 TEP faults, comparable to those in prior studies. While Ma et al. (2024)^60^ attained a higher average FDR by 2.42%, our approach excelled when excluding faults 3, 9, and 15, reaching 0.9674, the highest among the analyzed references.

## Conclusions

This study explored a new approach to fault detection in the Tennessee Eastman Process by combining a Siamese neural network architecture with transfer learning. We transformed the detection problem into an embedding similarity task using a pretrained fault diagnosis model as the backbone, enabling the model to differentiate between normal and faulty behavior more effectively. Our results demonstrate that the best-performing configuration achieved an F1-score of 91.41% on the test data set, with fault detection rates competitive with recent literature.

Our investigation addressed key challenges such as overfitting and stability. The stepwise freezing of convolutional layers proved critical in maintaining feature extraction quality while allowing for adaptability to the detection task. Despite the overall strong performance of our method, faults 3, 9, and 15 remain particularly difficult to detect. These faults are known to be subtle or slow-developing, as they are related to heat transfer phenomena. While our model achieved state-of-the-art performance for most faults, further refinements are needed to enhance detection for these complex scenarios. Future work could explore hybrid models that integrate temporal attention mechanisms and domain-specific physical constraints or explore the use of focal loss to enhance sensitivity to these cases.

It would benefit the literature if future works could also explore the impact of alternative distance measures on model performance, particularly in combination with modifications to the loss function. Furthermore, investigating the integration of the detection model with the diagnosis model in an ensemble framework could provide deeper insight into the full scope of the FDD problem. Finally, while our investigation focuses on the Tennessee Eastman Process data set due to its widespread use as a benchmark, applying our approach to other industrial data sets would provide valuable information about its generalization capabilities and potential for broader applications in practical fault detection scenarios.

## Supplementary Material



## Notes

Supplementary training and evaluation results, including training and validation curves (loss, F1-score, precision, recall), L2 gradient norm plots for all model configurations and stages, and output prediction histograms for the test of the best configuration (PDF)
