# Supplementary material for: A Diagnosis-Based Siamese Network for Fault Detection Through Transfer Learning
Source: J Chem Inf Model. 2025 Jun 30;65(13):6703–20. doi: 10.1021/acs.jcim.5c00809 (PMC12264958; doi:10.1021/acs.jcim.5c00809)
Supplement: Supplementary file 1 [file ci5c00809_si_001.pdf]

Supporting Information

A Diagnosis-based Siamese Network for Fault Detection Through Transfer Learning

João G. Neto[1], Karla Figueiredo[2], João B. P. Soares[3], Amanda L. T. Brandão[1,]*

1 Department of Chemical and Materials Engineering, Pontifical Catholic University of Rio de Janeiro, 225, Marquês de São Vicente Street, Gávea, Rio de Janeiro, RJ, Brazil, 22451-900.

2 Department of Computer Science, Rio de Janeiro State University, 524, Rector João Lyra Filho Pavilion, 6th floor, Maracanã, Rio de Janeiro, RJ, Brazil, 20550-013.

3 Department of Chemical Engineering, University of Alberta, 9211, 116 Street, Edmonton, Alberta, Canada, T6G 1H9.

*Corresponding Author

Email: amanda.lemette@puc-rio.br

Stage 1 Supplementary Figures - Effects of training dataset size and baseline

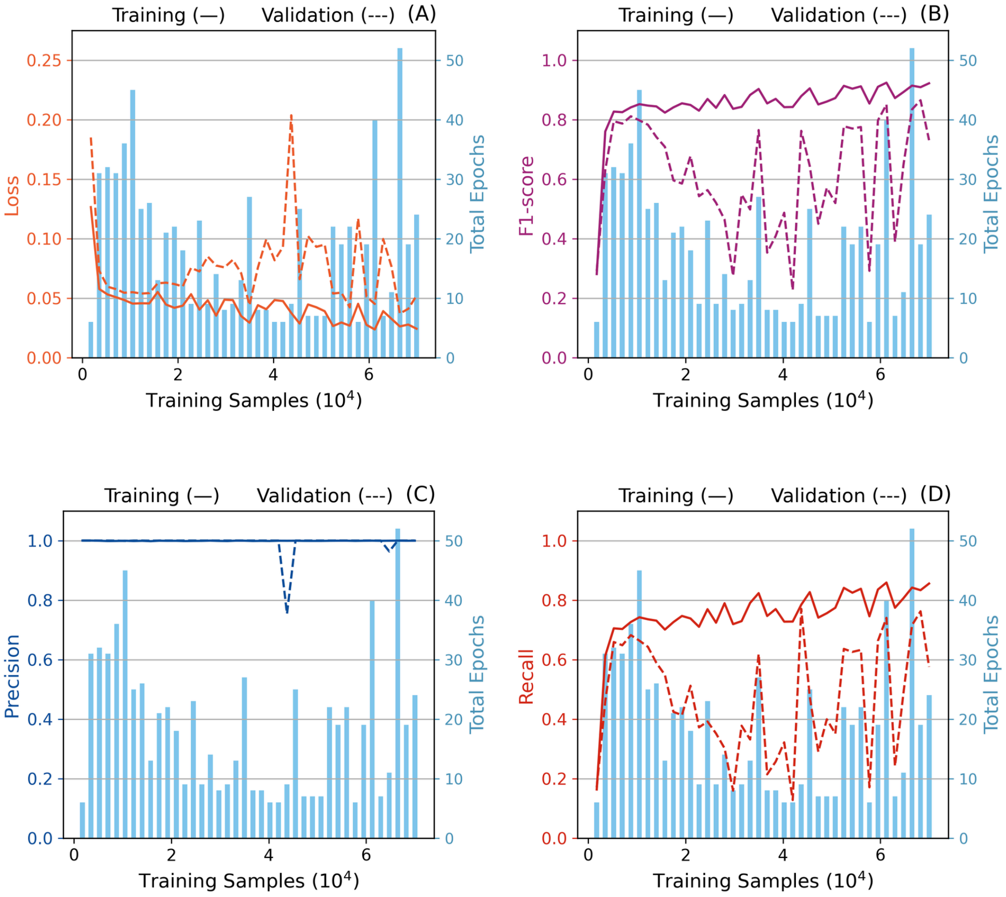

Figure S1. Loss (A), F1-Score (B), Precision (C) and Recall (D) learning curves with early stopping.

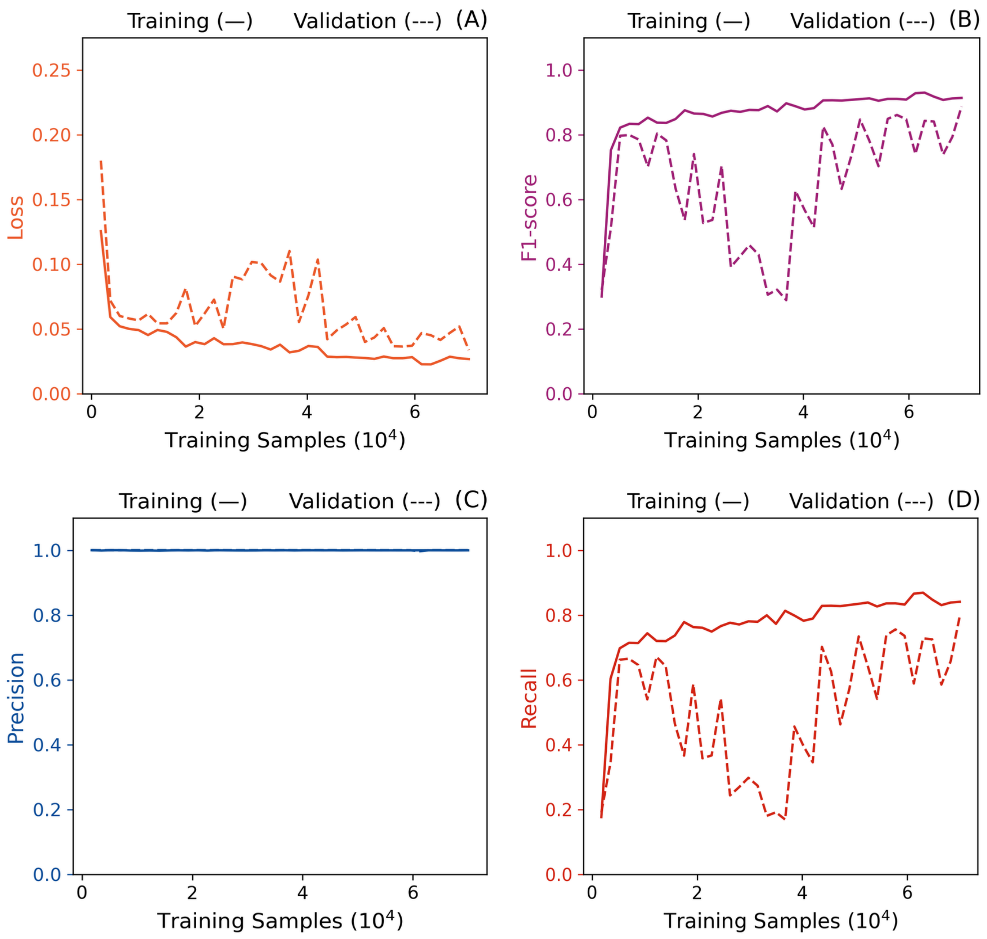

Figure S2. Loss (A), F1-Score (B), Precision (C) and Recall (D) learning curves with 30 epochs.

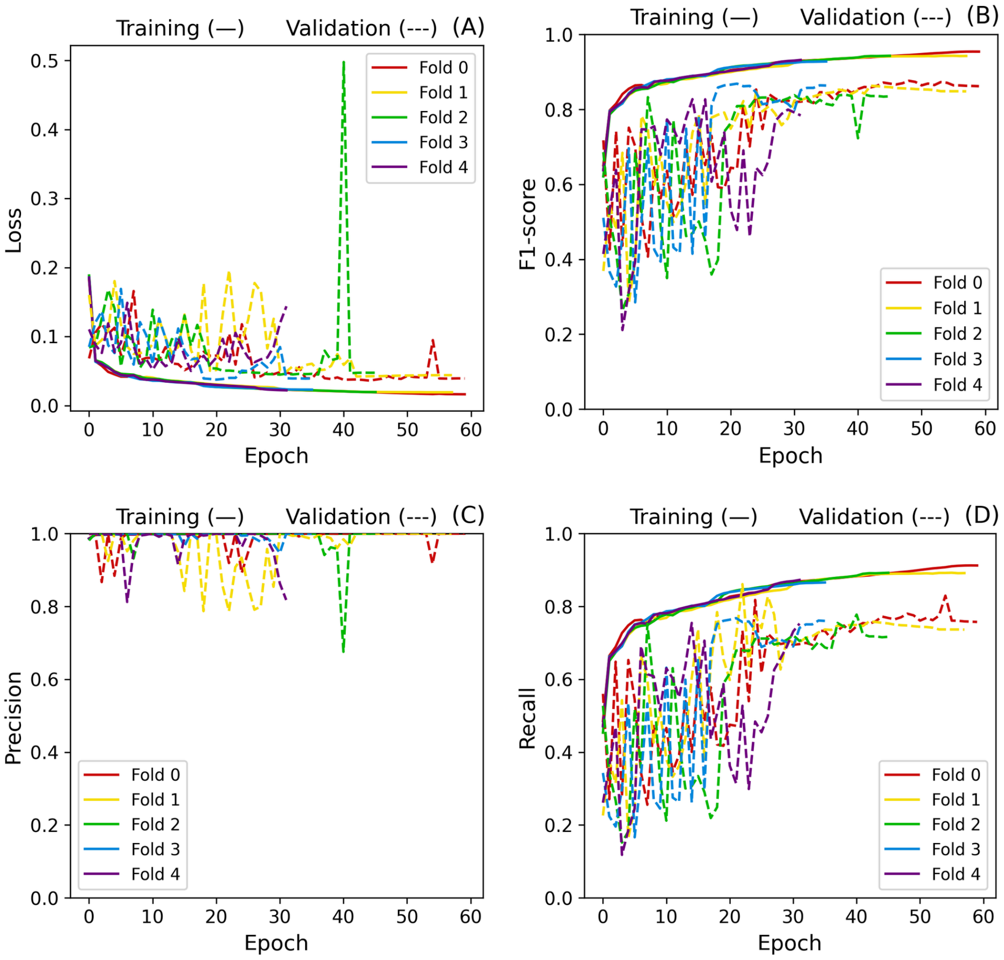

Figure S3. Baseline cross-validation Loss (A), F1-Score (B), Precision (C) and Recall (D) curves.

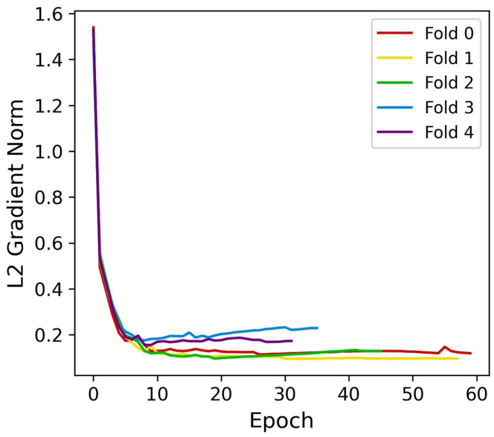

Figure S4. Baseline L2 gradient norm.

Stage 2 Supplementary Figures - Model stability and overfitting mitigation

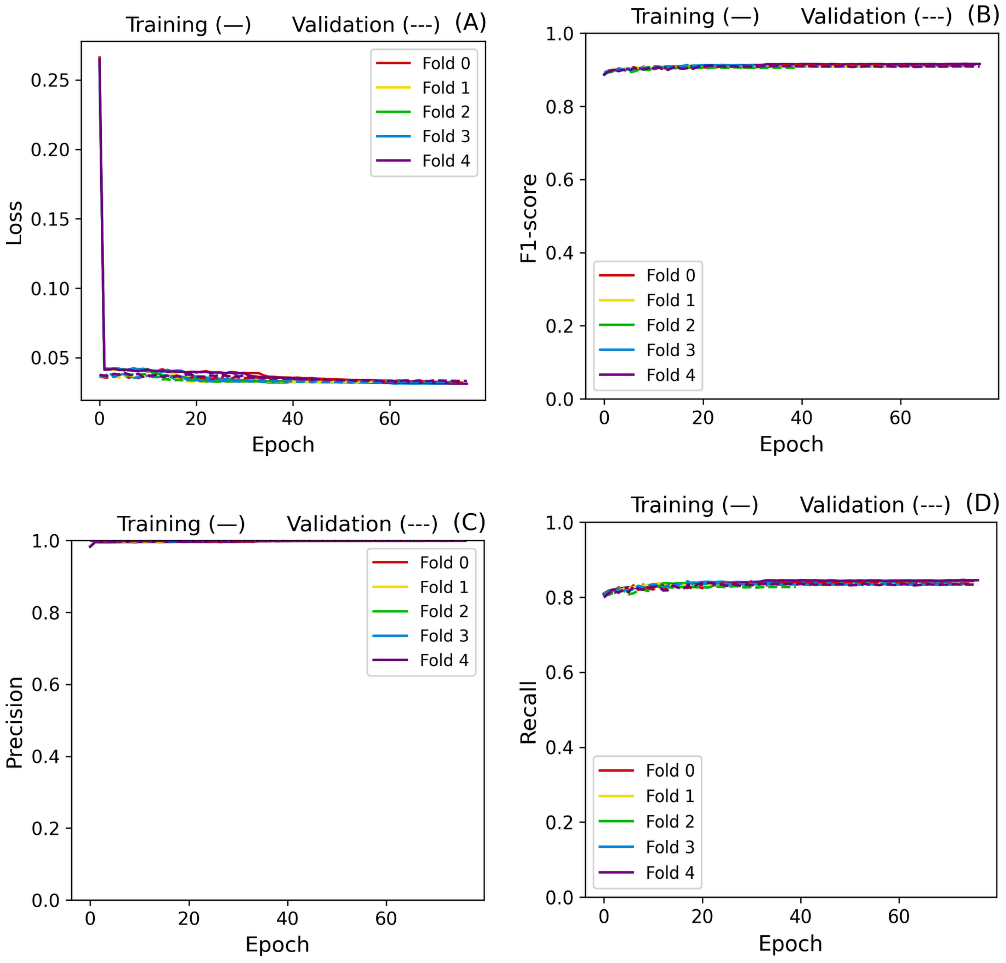

Figure S5. Cross-validation Loss (A), F1-Score (B), Precision (C) and Recall (D) curves after freezing convolutional layers.

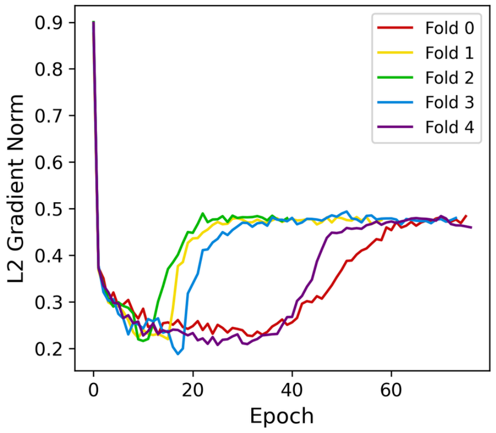

Figure S6. L2 gradient norm after freezing convolutional layers.

Stage 3 Supplementary Figures - Model improvement investigation

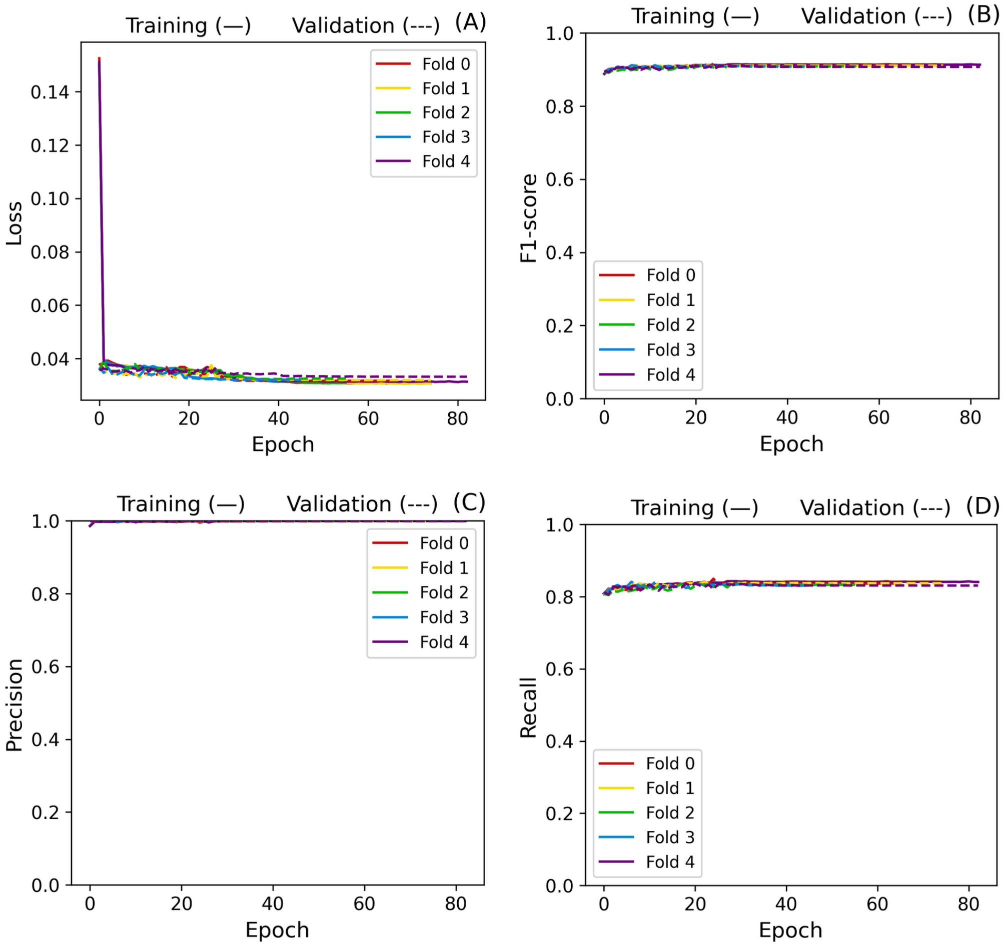

Figure S7. Cross-validation Loss (A), F1-Score (B), Precision (C) and Recall (D) curves of model with reduced dropout rate (30 %).

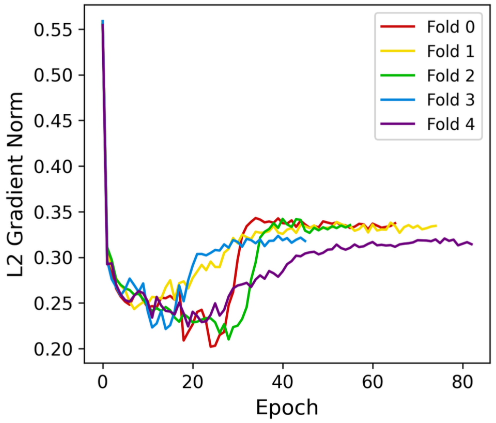

Figure S8. L2 gradient norm of model with reduced dropout rate (30 %).

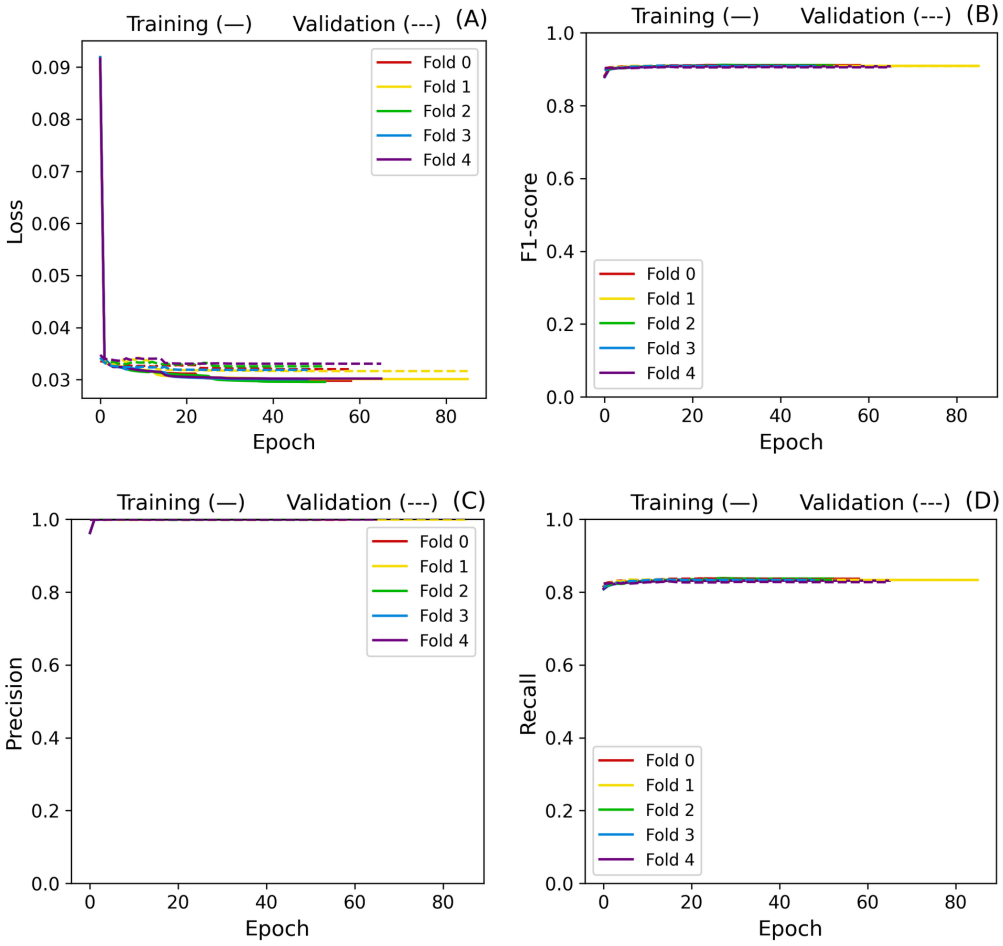

Figure S9. Cross-validation Loss (A), F1-Score (B), Precision (C) and Recall (D) curves of model with no dropout layer.

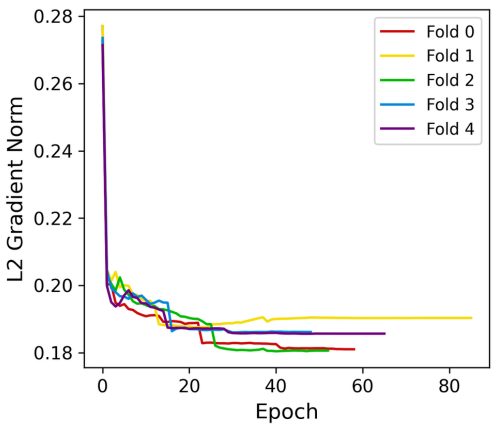

Figure S10. L2 gradient norm of model with no dropout layer.

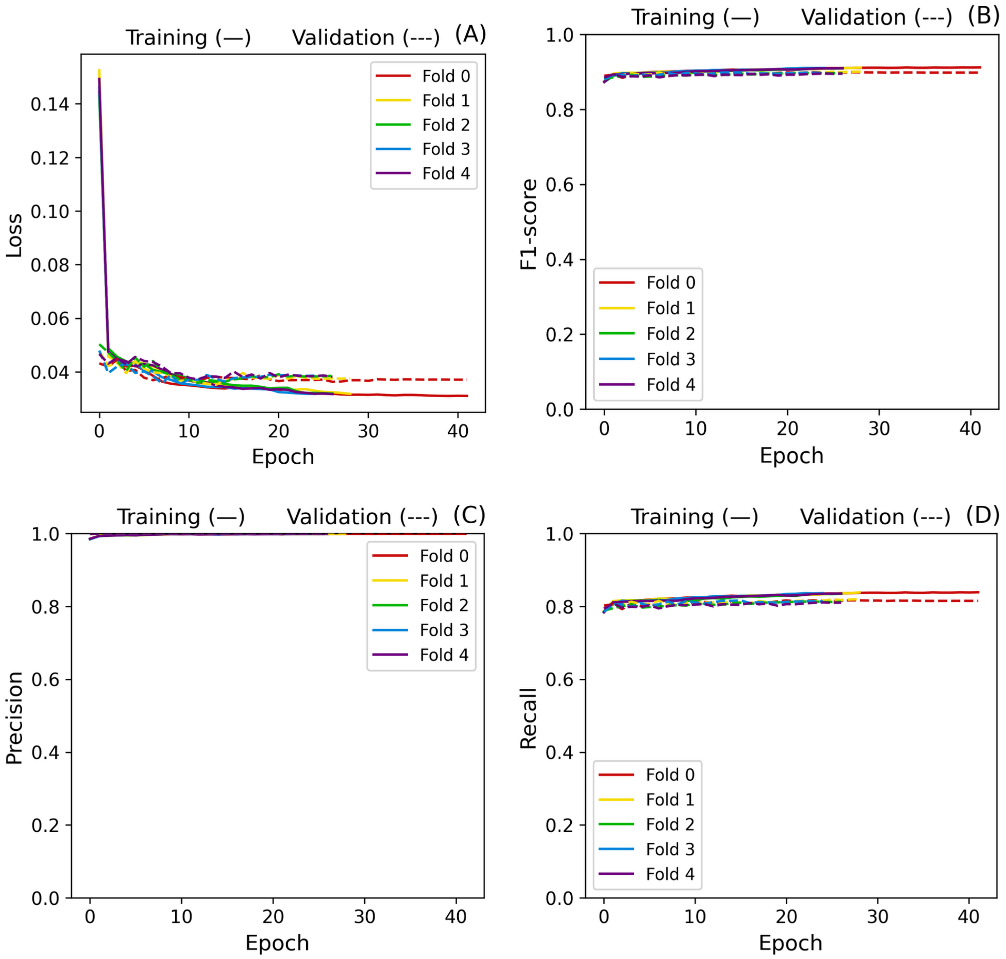

Figure S11. Cross-validation Loss (A), F1-Score (B), Precision (C) and Recall (D) curves of model with additional dense layer with 512 neurons.

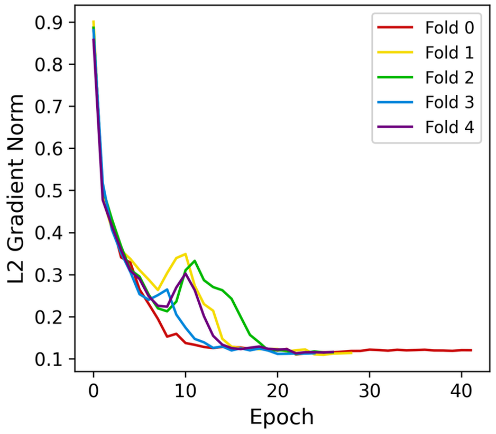

Figure S12. L2 gradient norm of model with additional dense layer with 512 neurons.

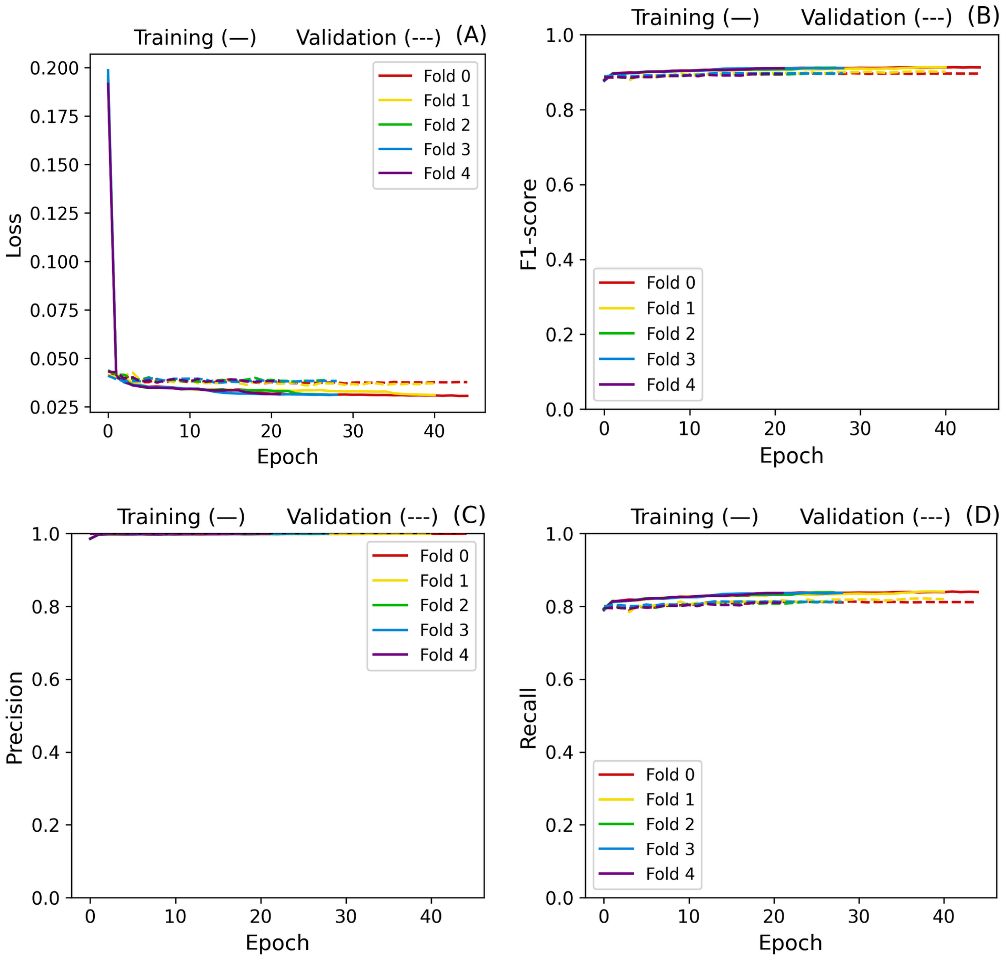

Figure S13. Cross-validation Loss (A), F1-Score (B), Precision (C) and Recall (D) curves of model with additional dense layer with 1024 neurons.

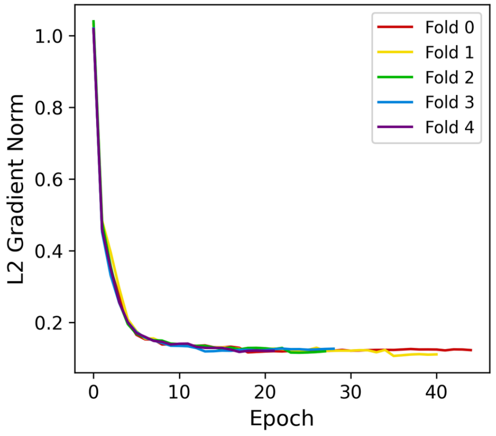

Figure S14. L2 gradient norm of model with additional dense layer with 1024 neurons.

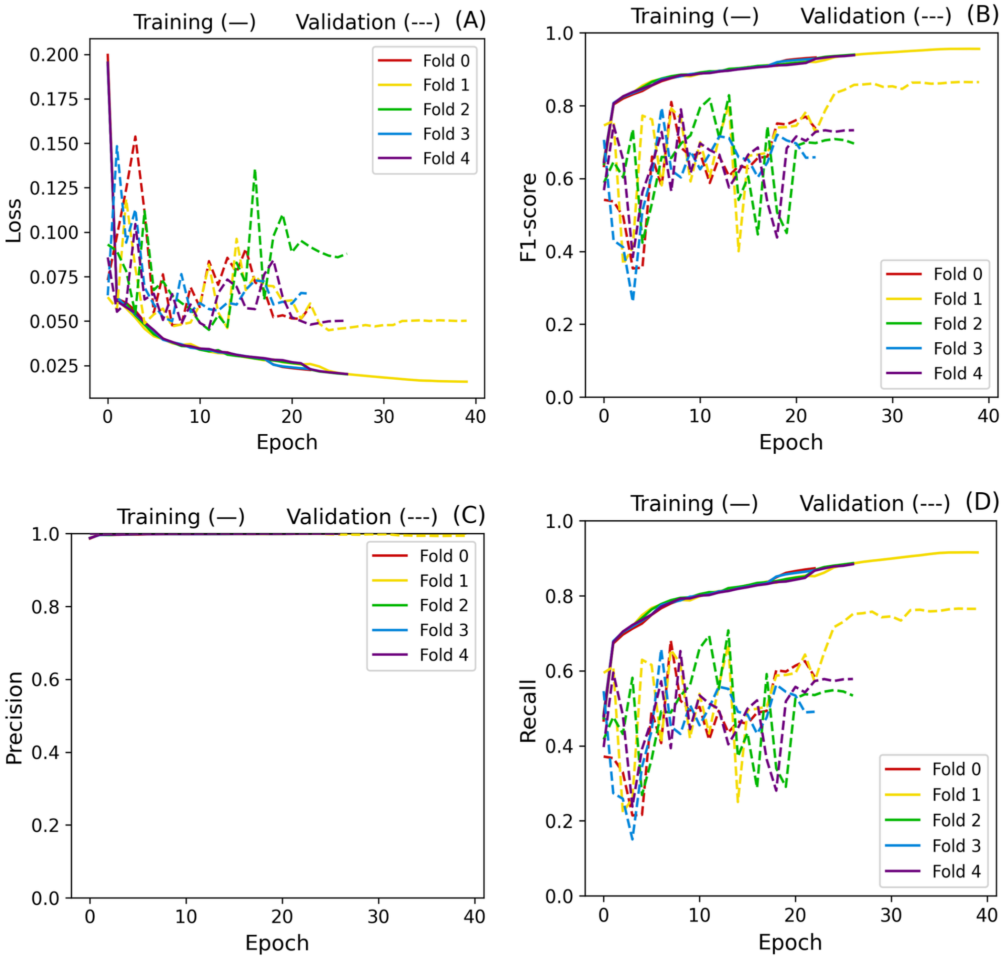

Figure S15. Cross-validation Loss (A), F1-Score (B), Precision (C) and Recall (D) curves after freezing Group 1.

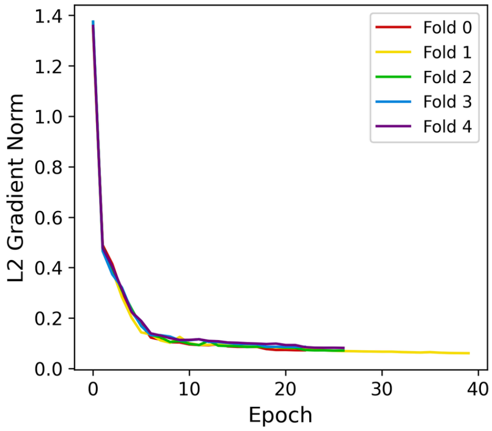

Figure S16. L2 gradient norm after freezing Group 1.

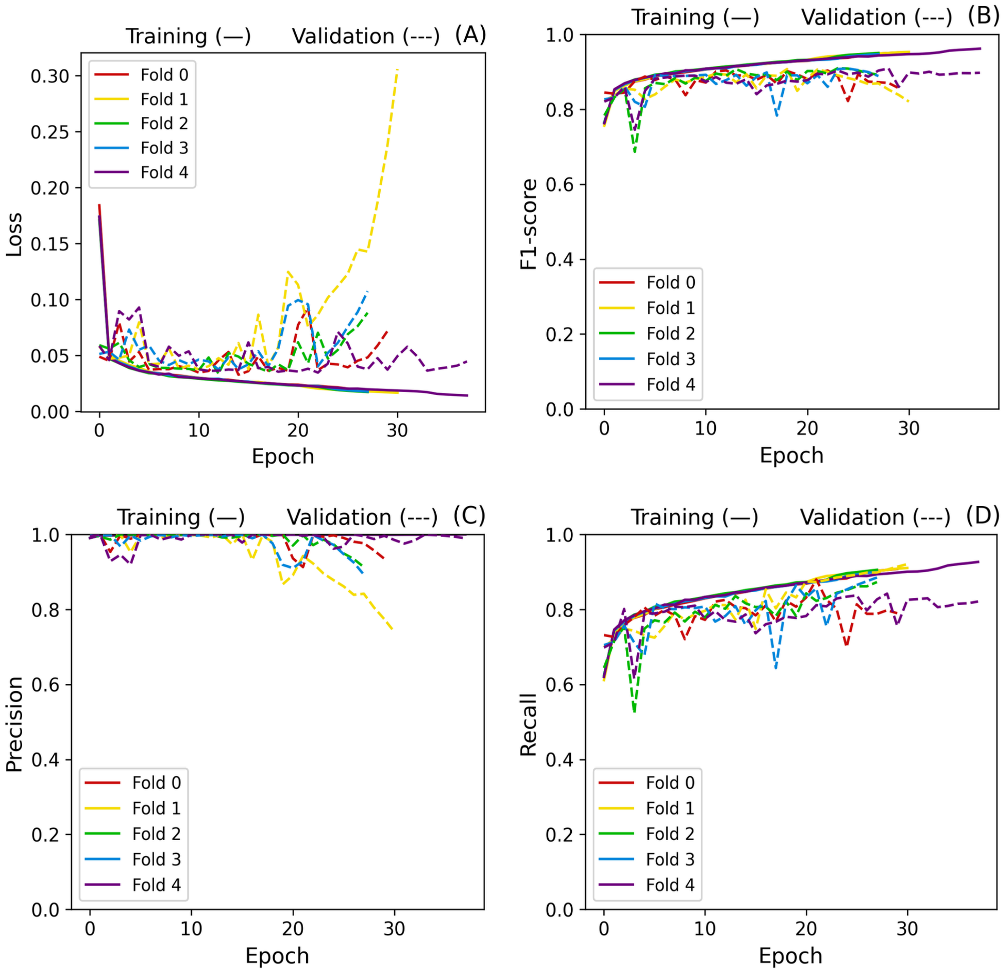

Figure S17. Cross-validation Loss (A), F1-Score (B), Precision (C) and Recall (D) curves after freezing Group 2.

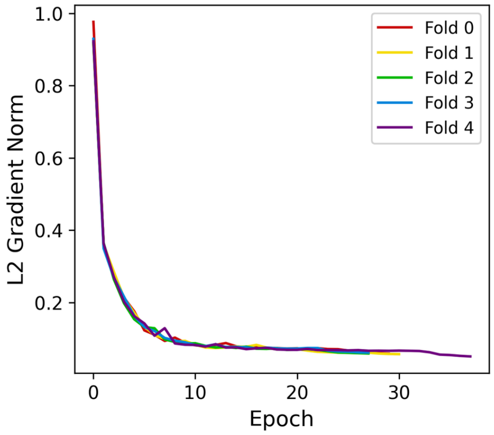

Figure S18. L2 gradient norm after freezing Group 2.

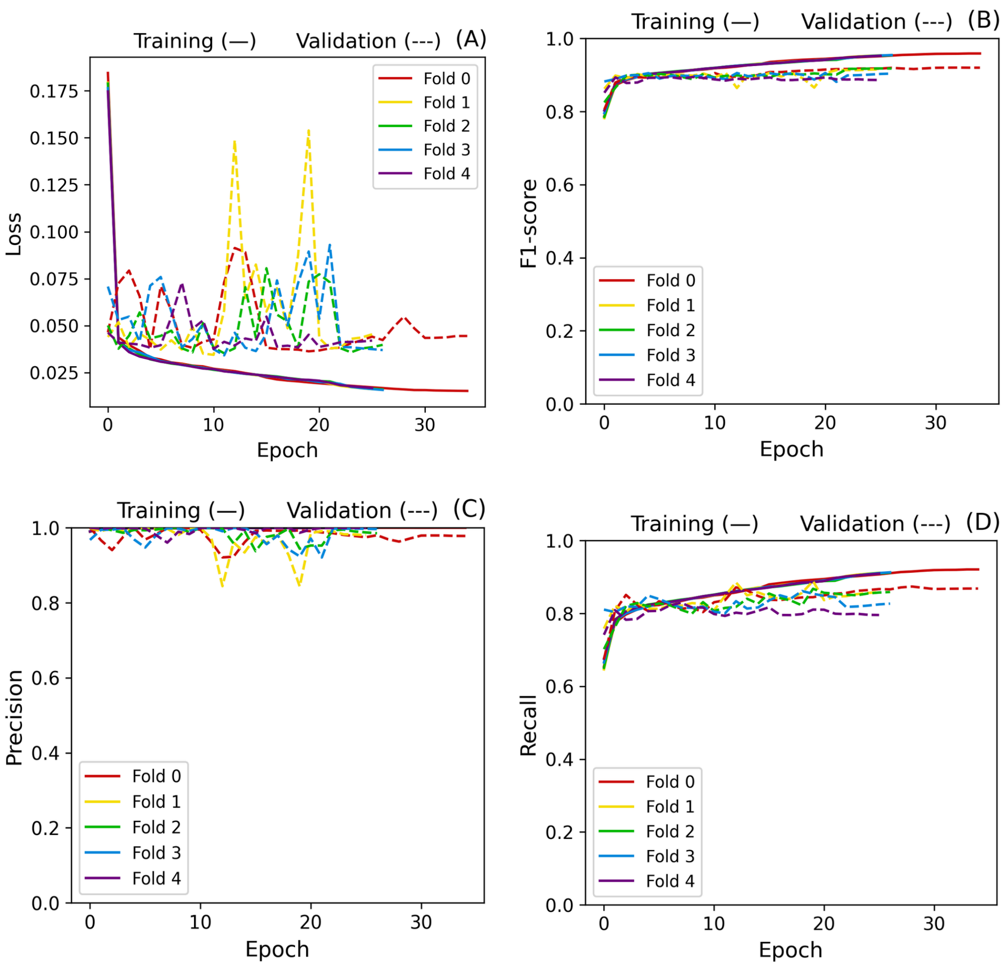

Figure S19. Cross-validation Loss (A), F1-Score (B), Precision (C) and Recall (D) curves after freezing Group 3.

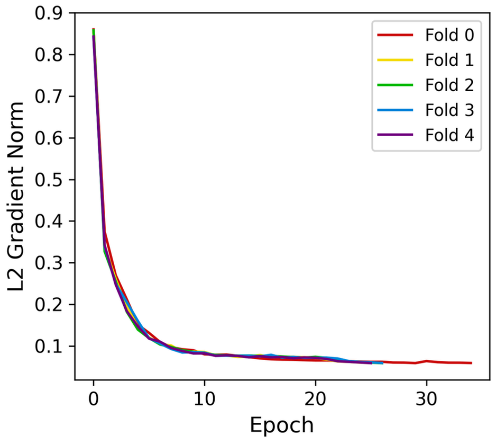

Figure S20. L2 gradient norm after freezing Group 3.

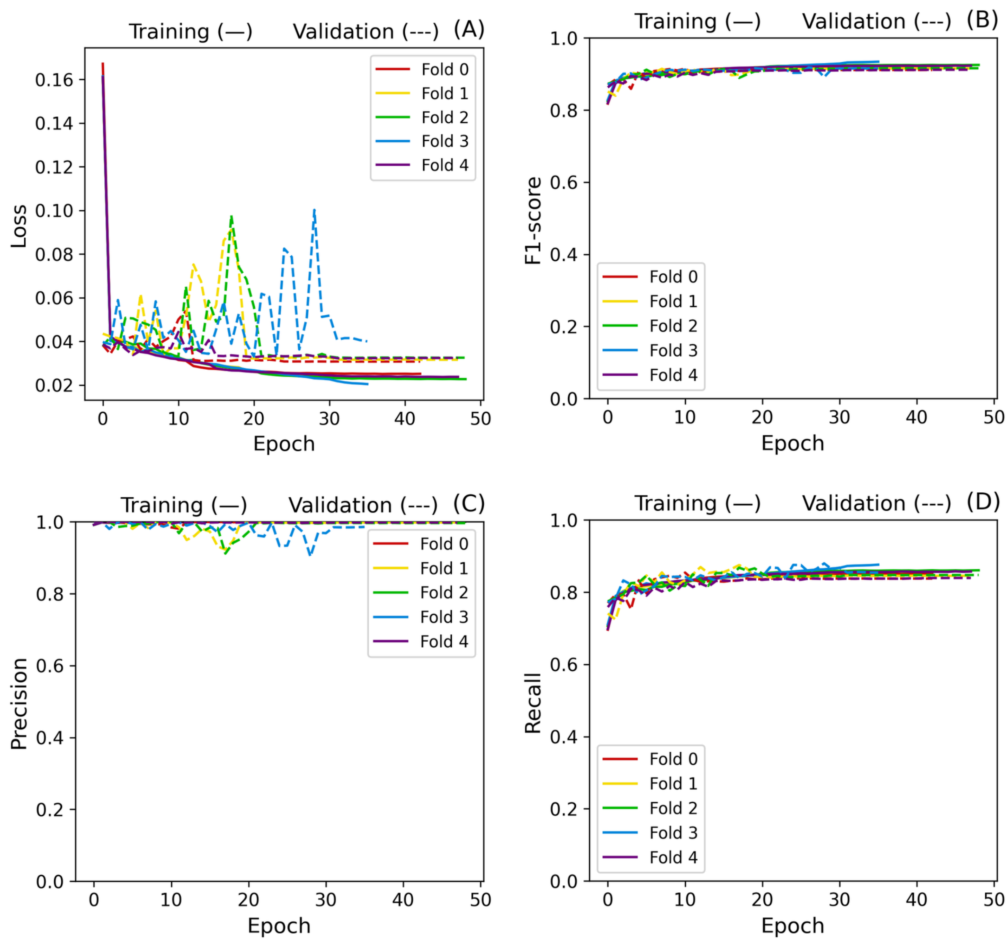

Figure S21. Cross-validation Loss (A), F1-Score (B), Precision (C) and Recall (D) curves after freezing Group 4.

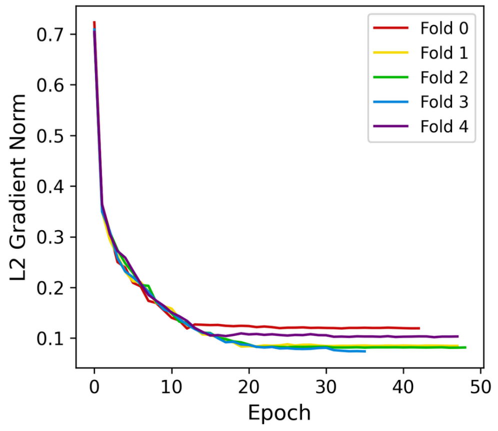

Figure S22. L2 gradient norm after freezing Group 4.

Stage 4 Supplementary Figures - Best configuration results

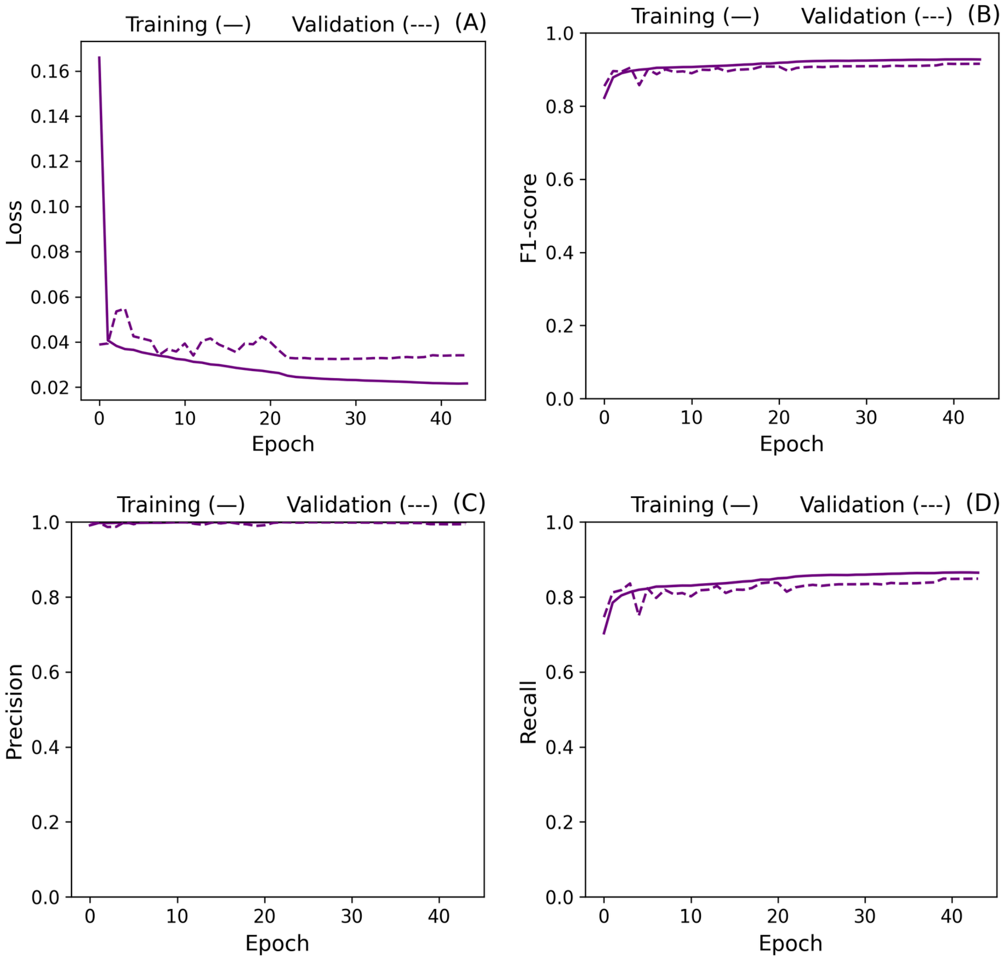

Figure S23. Best model's holdout (A), F1-Score (B), Precision (C) and Recall (D) curves.

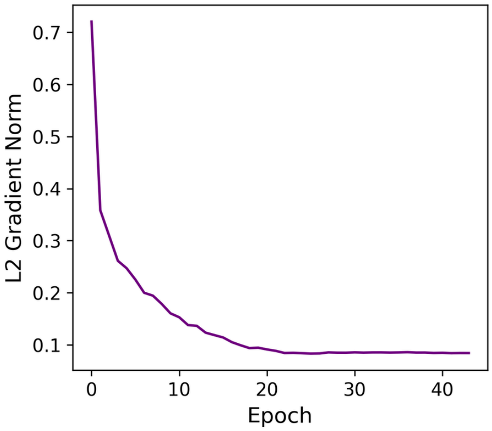

Figure S24. Best model's holdout L2 gradient norm.

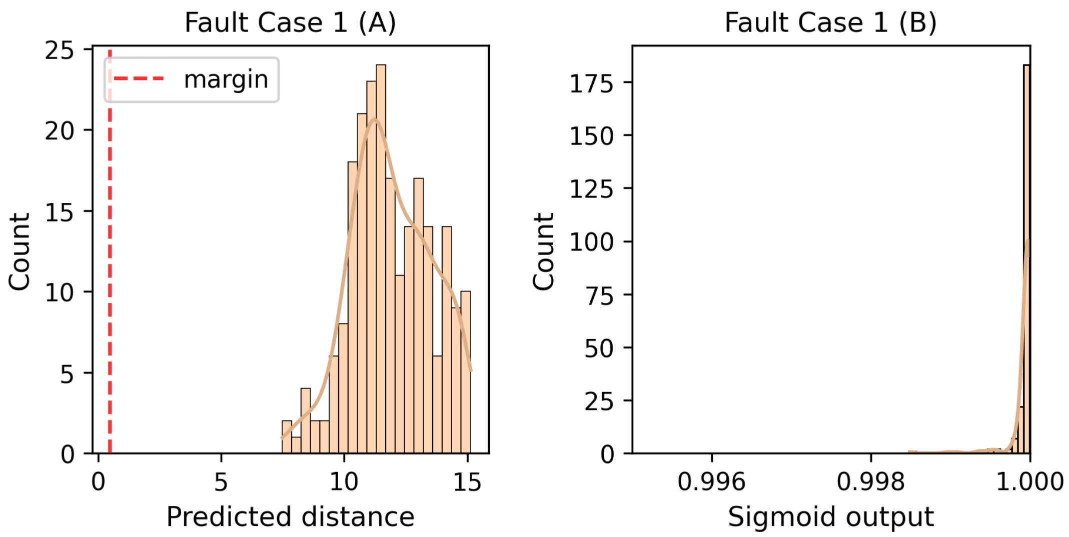

Figure S25. Test predicted distance (A) and sigmoid output (B) distributions of fault case 1.

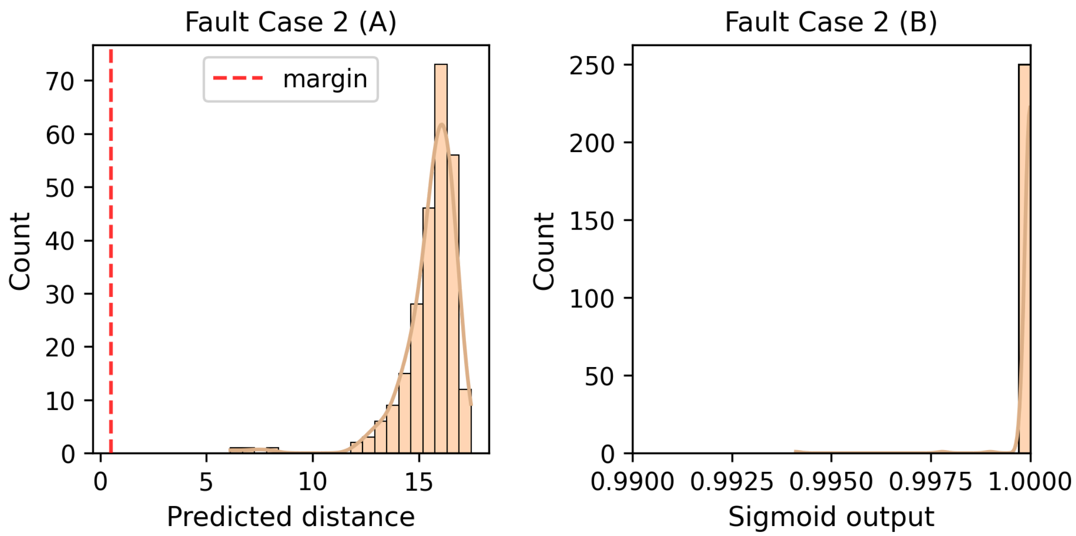

Figure S26. Test predicted distance (A) and sigmoid output (B) distributions of fault case 2.

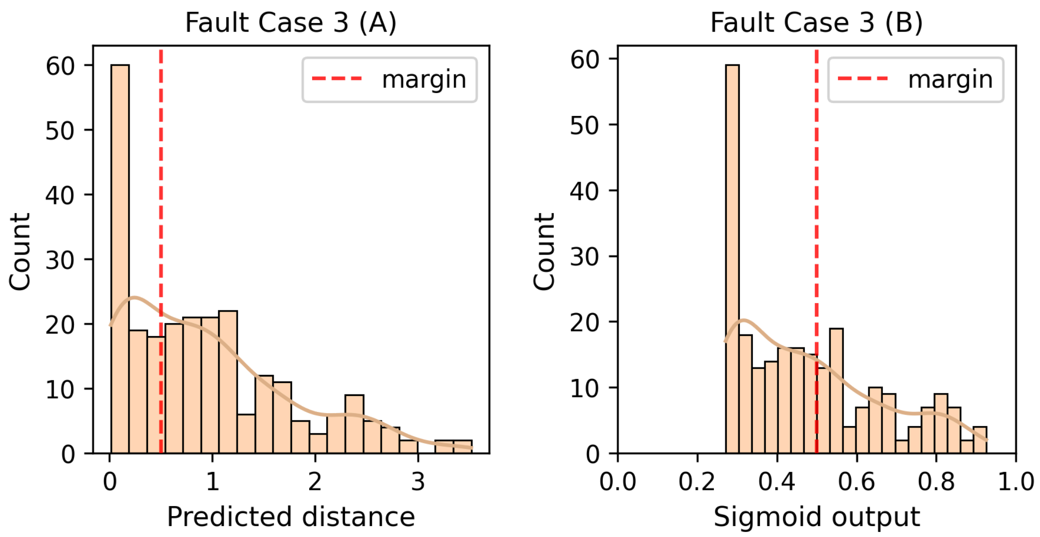

Figure S27. Test predicted distance (A) and sigmoid output (B) distributions of fault case 3.

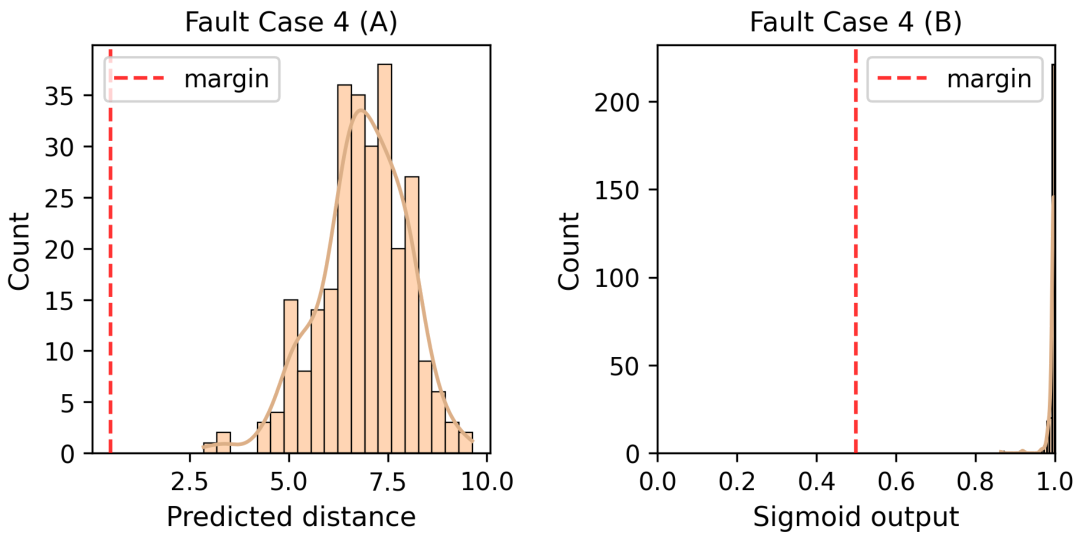

Figure S28. Test predicted distance (A) and sigmoid output (B) distributions of fault case 4.

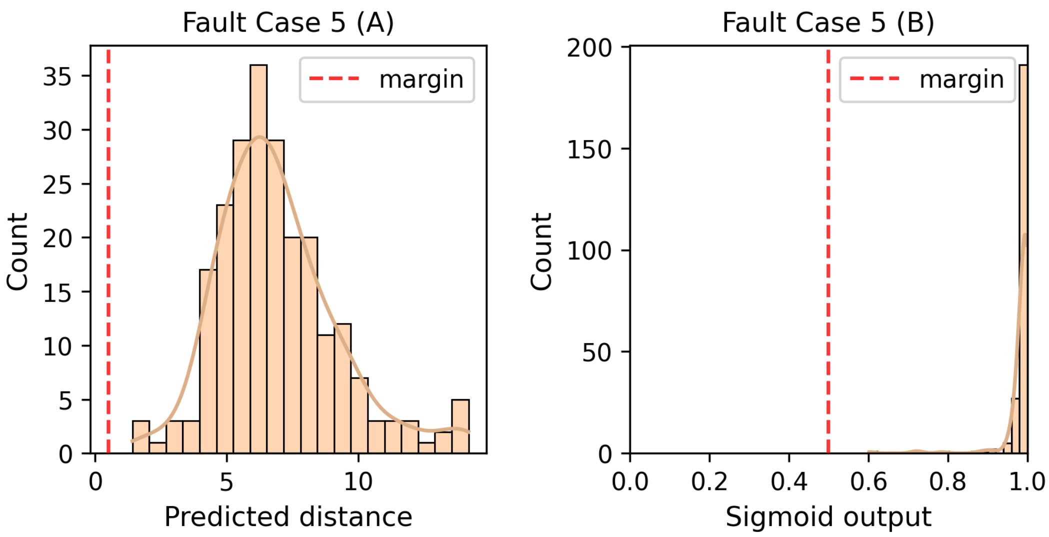

Figure S29. Test predicted distance (A) and sigmoid output (B) distributions of fault case 5.

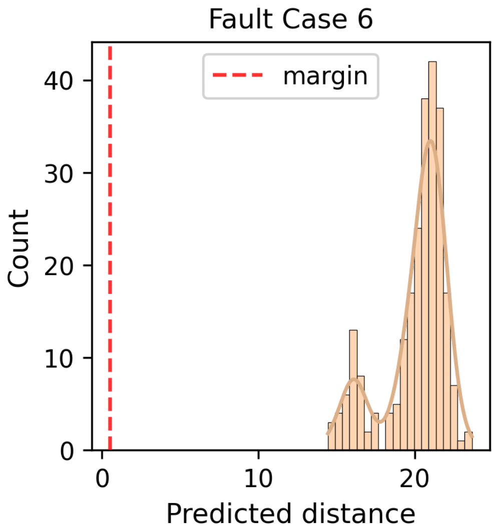

Figure S30. Test predicted distance distributions of fault case 6. The sigmoid transformation could not be represented graphically as a distribution because all values tend to 1.0.

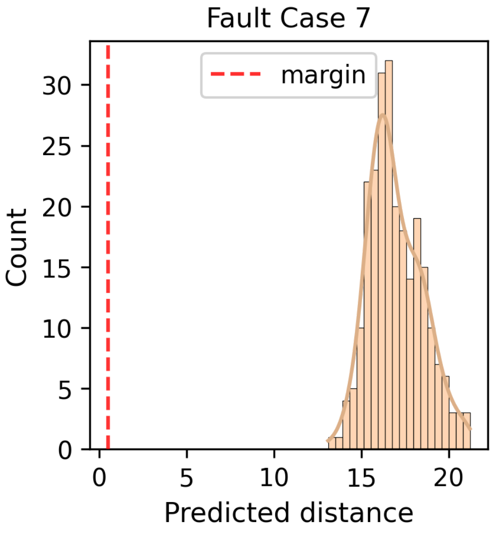

Figure S31. Test predicted distance distributions of fault case 7. The sigmoid transformation could not be represented graphically as a distribution because all values tend to 1.0.

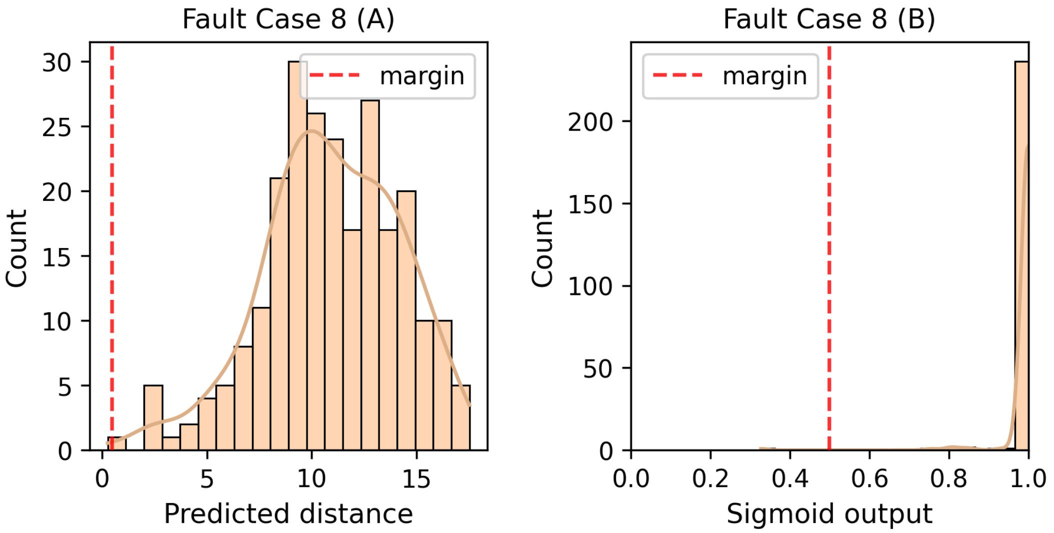

Figure S32. Test predicted distance (A) and sigmoid output (B) distributions of fault case 8.

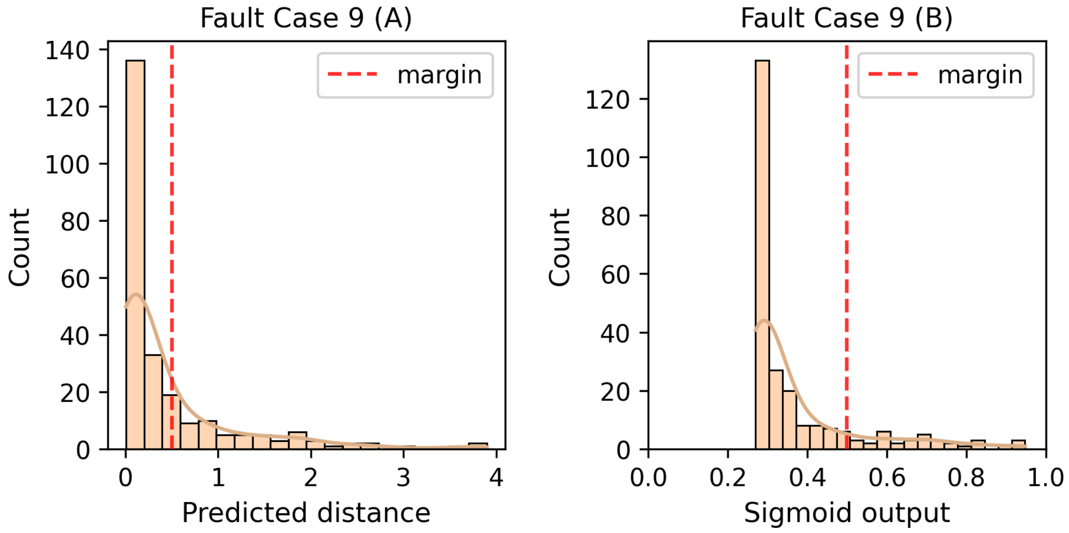

Figure S33. Test predicted distance (A) and sigmoid output (B) distributions of fault case 9.

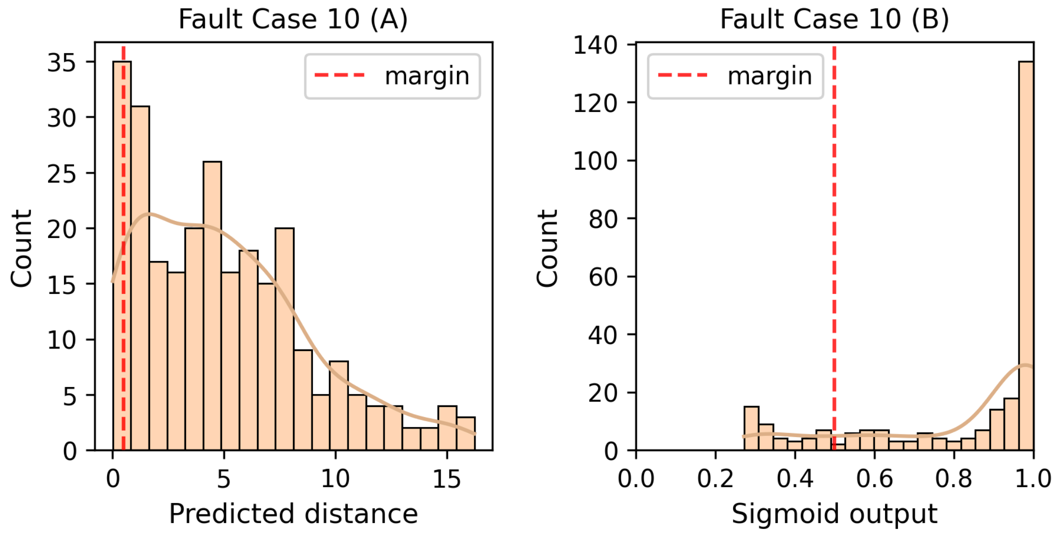

Figure S34. Test predicted distance (A) and sigmoid output (B) distributions of fault case 10.

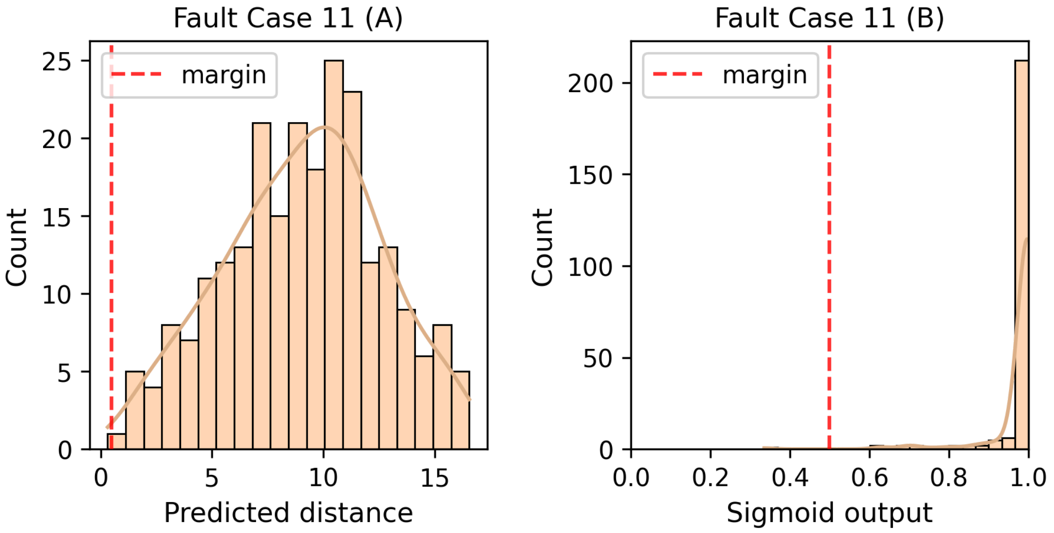

Figure S35. Test predicted distance (A) and sigmoid output (B) distributions of fault case 11.

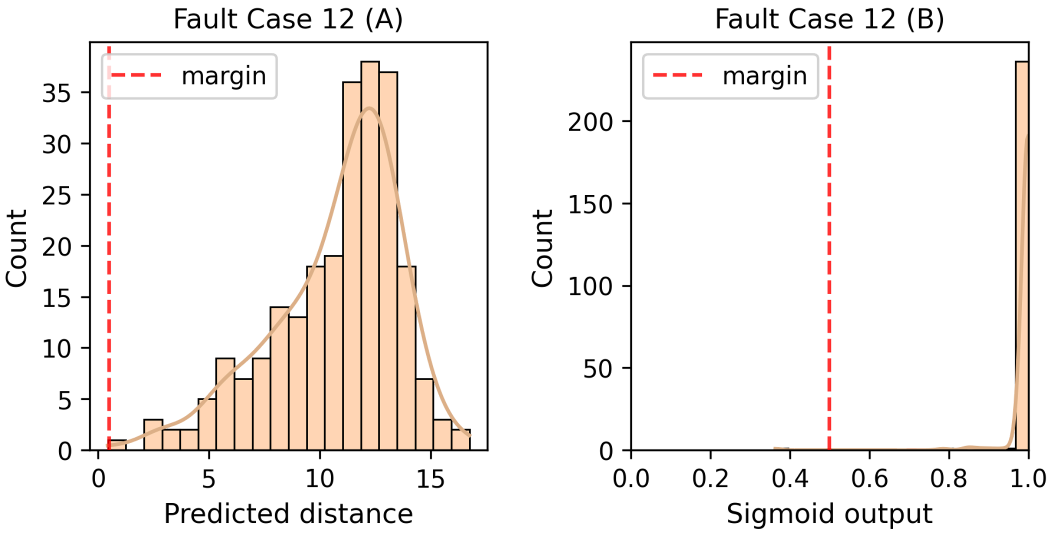

Figure S36. Test predicted distance (A) and sigmoid output (B) distributions of fault case 12.

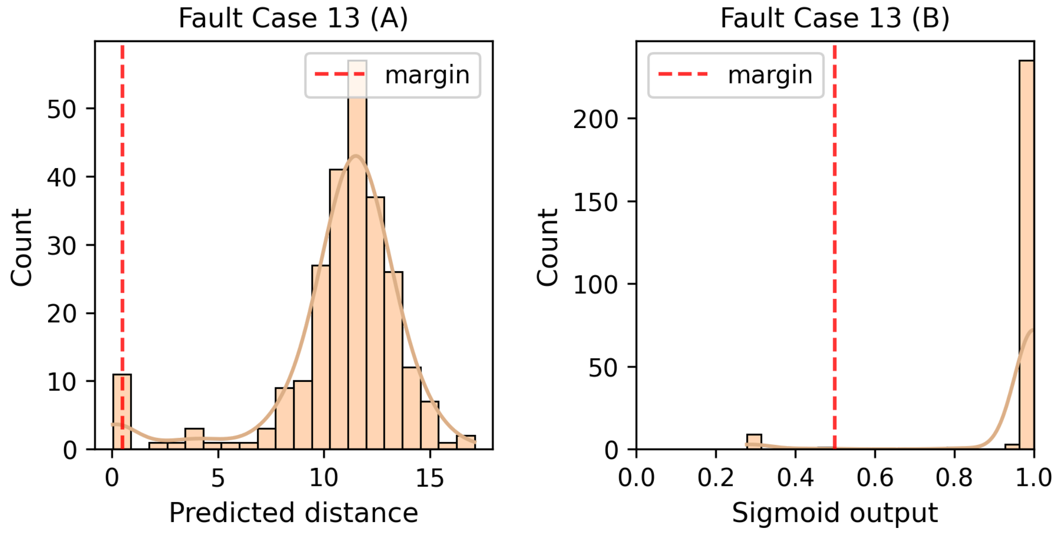

Figure S37. Test predicted distance (A) and sigmoid output (B) distributions of fault case 13.

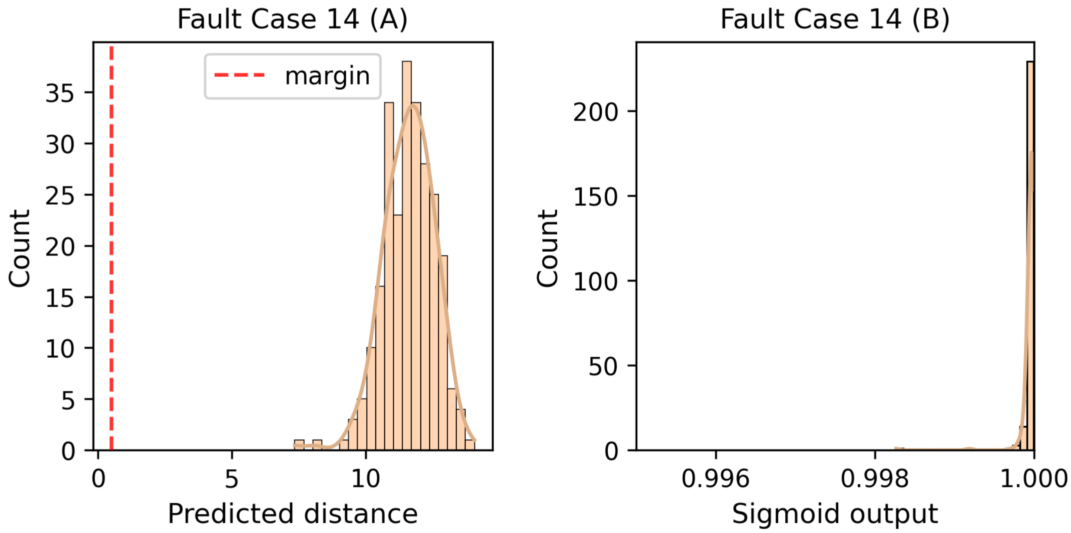

Figure S38. Test predicted distance (A) and sigmoid output (B) distributions of fault case 14.

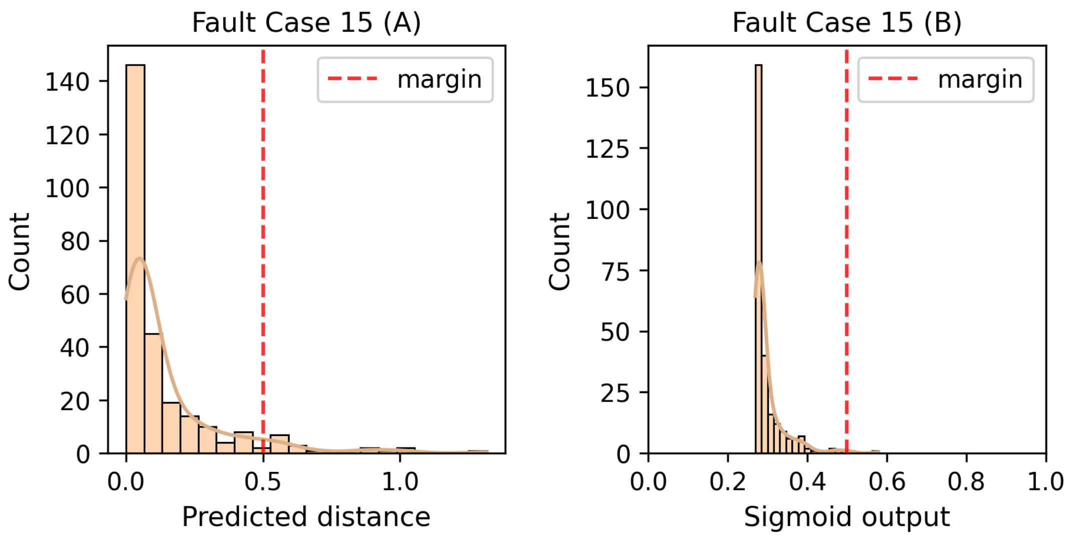

Figure S39. Test predicted distance (A) and sigmoid output (B) distributions of fault case 15.

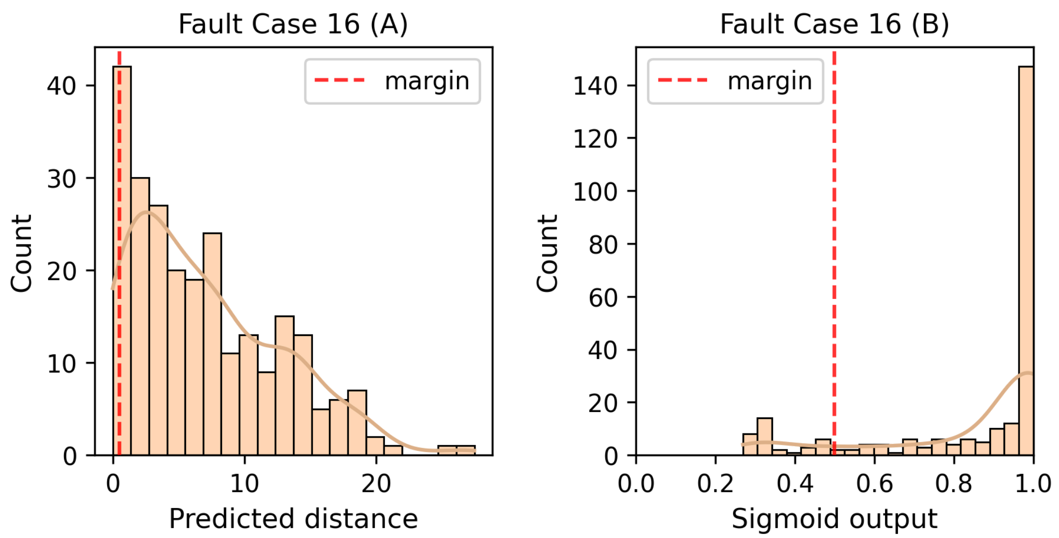

Figure S40. Test predicted distance (A) and sigmoid output (B) distributions of fault case 16.

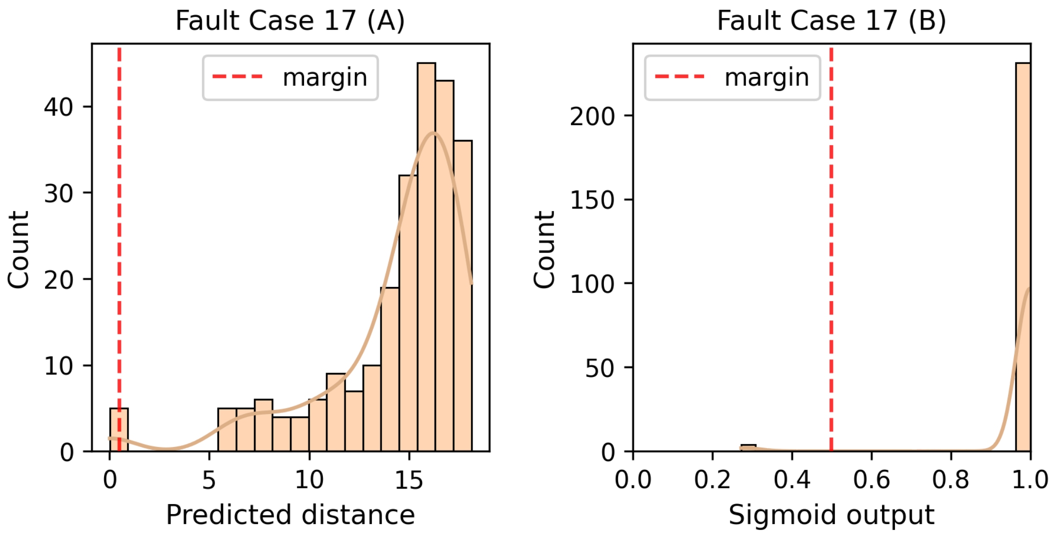

Figure S41. Test predicted distance (A) and sigmoid output (B) distributions of fault case 17.

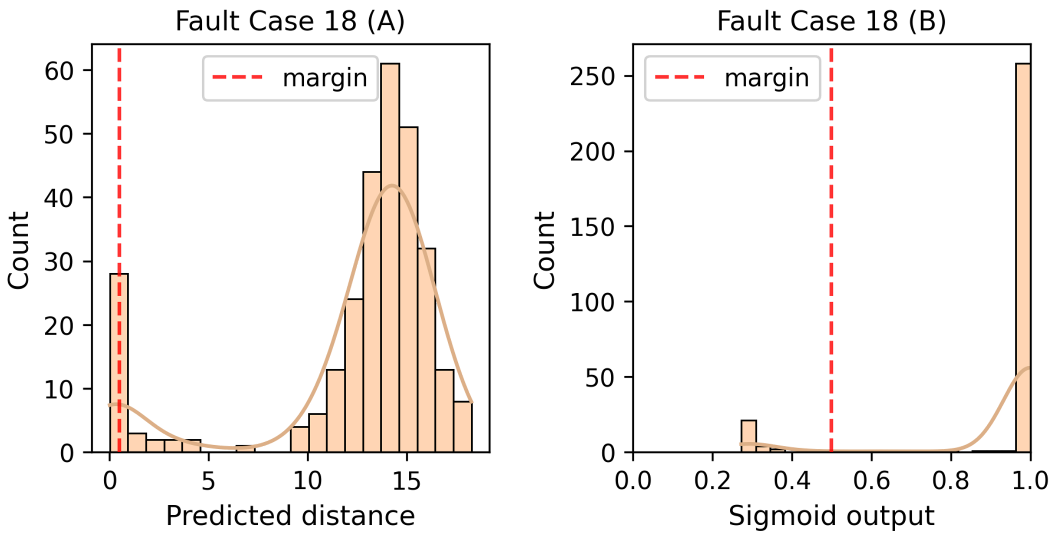

Figure S42. Test predicted distance (A) and sigmoid output (B) distributions of fault case 18.

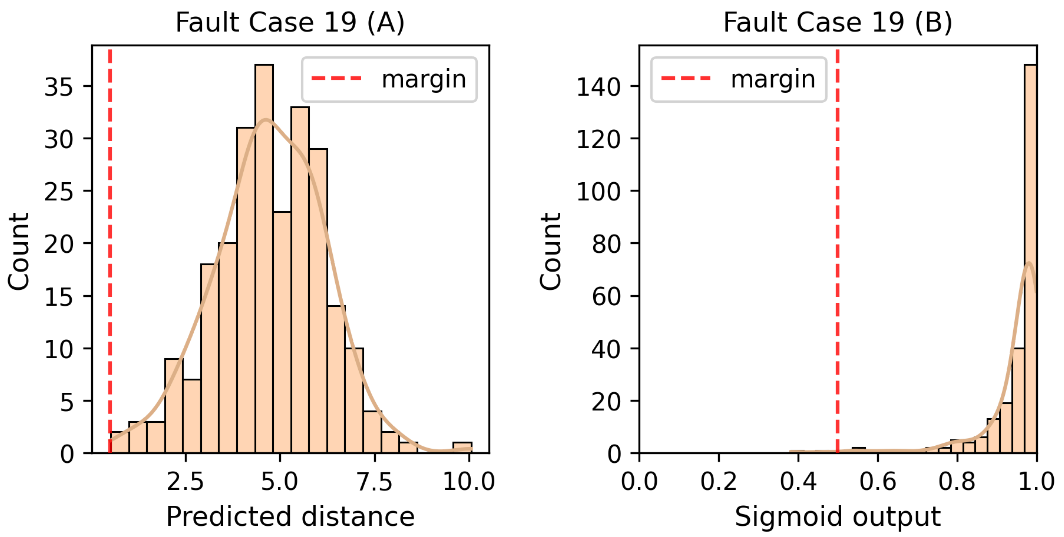

Figure S43. Test predicted distance (A) and sigmoid output (B) distributions of fault case 19.

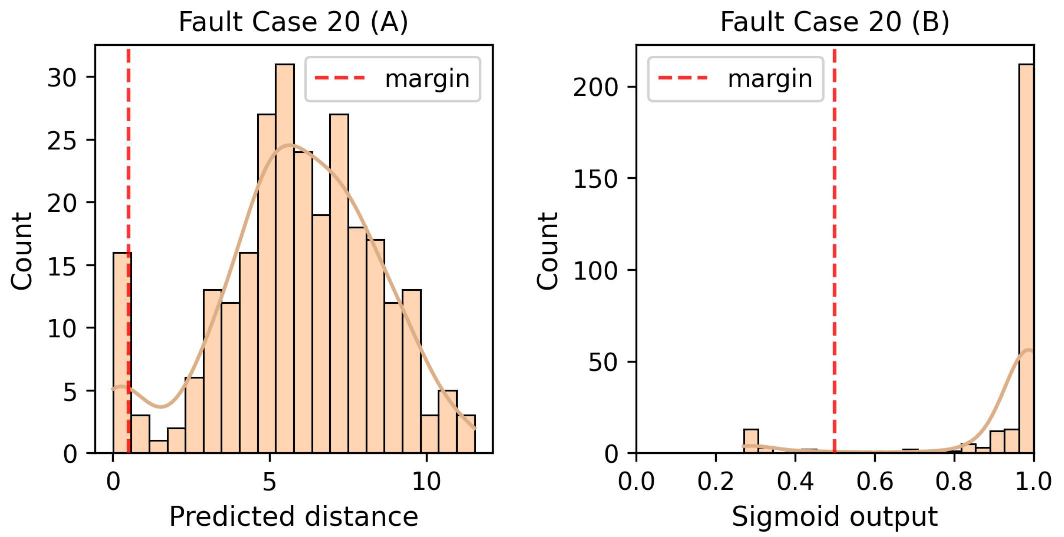

Figure S44. Test predicted distance (A) and sigmoid output (B) distributions of fault case 20.
